# Nanobiotechnology-driven advances in sonodynamic therapy: unlocking the potential of sonosensitizers in clinical translation

**DOI:** 10.1039/d5ra03591k

**Published:** 2026-01-28

**Authors:** Zhaokui Zeng, Tian Tian, Jia Liu, Letian Bai, Jun Zhang, Changhong Nie, Bo Liu, Chuanpin Chen, Wei Lu

**Affiliations:** a School of Pharmaceutical Sciences, Jishou University Jishou 416000 China; b Department of Pharmacy, Xiangya School of Pharmaceutical Sciences, Central South University Changsha 410000 China ccpin2000@hotmail.com; c Department of General Surgery, The Second Xiangya Hospital, Central South University Changsha 410000 China; d Department of Cardiovascular Surgery, The Quzhou Affiliated Hospital of Wenzhou Medical University, Quzhou People's Hospital Quzhou 324000 China luwei@wmu.edu.cn; e Key Laboratory of Medicinal Resources Chemistry and Pharmacology in Wuling Mountainous of Hunan Province College Jishou 416000 China

## Abstract

Sonodynamic therapy (SDT) is an emerging, non-invasive modality to cancer treatment, notable for its ability to overcome the limited tissue penetration of photodynamic therapy (PDT) while delivering enhanced precision and reduced adverse effects. This distinctive advantage has positioned SDT as a highly compelling approach within the biomedical landscape. Central to the success of SDT is the development of sonosensitizers, whose design and functionality critically influence therapeutic efficacy. This review discussed the fundamental principles and mechanism of SDT. It highlighted recent advances in nanobiotechnology-based sonosensitizers, including inorganic sonosensitizers, organic sonosensitizers, inorganic–organic hybrid sonosensitizers and other sonosensitizers, and also indicated the specific designs and potential challenges of sonosensitizers. Furthermore, this review outlined the obstacles that need to be overcome for the successful clinical translation and application of SDT. In conclusion, SDT represents a promising strategy for cancer therapy, with sonosensitizers expected to play a pivotal role in integrating diagnostics, enhancing drug delivery, and enabling multimodal treatment approaches.

## Abstract 2

Schematic illustration of the sonosensitizers used for SDT.

## Graphical abstract

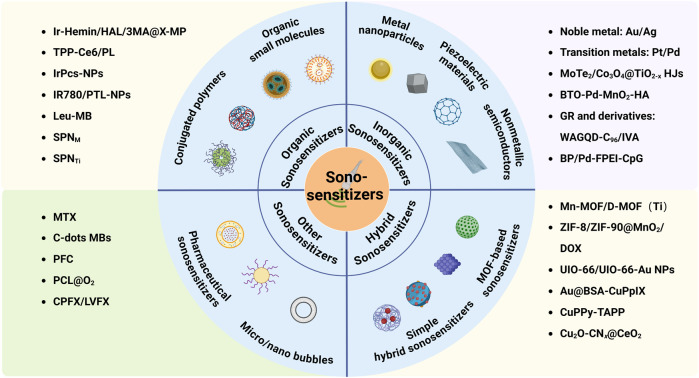

## 1. Introduction

Ultrasound (US), a mechanical wave with frequencies above the audible range of the human ear (20 kHz),^1^ has been widely utilized in areas such as clinical diagnostic imaging, transdermal drug delivery, thrombosis treatment and localized drug activation.^2–4^ Sonodynamic therapy (SDT) is a novel non-invasive treatment modality that employs US to induce cavitation within the target tissue. The energy and microenvironmental effects produced by these US-mediated cavitation events are then harnessed by sonosensitizers to generate cytotoxic reactive oxygen species (ROS), thereby achieving localized therapeutic effects. Notably, some nanostructured sonosensitizers can lower the cavitation threshold and enhance the efficiency of this energy conversion process.^5–7^ Unlike photodynamic therapy (PDT), which is inherently limited by a shallow tissue penetration depth (<1 cm) due to absorption and scattering by components such as water, melanin, fat, and collagen,^8–11^ SDT can penetrate more than 10 cm into soft tissues.^12^ This unique advantage of minimal tissue scattering allows for efficient activation of nano-sonosensitizers in deep-seated tumor lesions with high precision while minimizing damage to surrounding healthy tissue.^9^ Moreover, SDT's sonosensitizers often exhibit multifunctionality, such as therapeutic enhancement and imaging capabilities, which could enable the integration of diagnosis, monitoring and treatment, offering great promise for clinical applications.^13^ An increasing number of studies, with about 2026 papers indexed in Pubmed as of November 6, 2025 have demonstrated the potential of SDT as an effective treatment for cancer,^14^ neurodegenerative diseases,^15^ and cardiovascular conditions.^16^

The therapeutic efficacy of SDT is intrinsically tied to the performance of sonosensitizers. Sonosensitizers are acoustically responsive materials, their stability, ability to accumulate in target tissues and acoustic activity are closely linked to the therapeutic effect of SDT. Traditional sonosensitizers, such as organic small molecules (*e.g.*, hematoporphyrin), often suffer from instability, low bioavailability, and inadequate ROS production, resulting in suboptimal tumor treatment.^17,18^ As a result, developing sonosensitizers with greater stability, enhanced acoustic responsiveness, and higher ROS yield has become a central research priority in this field. In recent years, advances in nanobiotechnology have significantly driven the innovation and optimization of conventional sonosensitizers. Traditional sonosensitizers, such as small organic molecule,^8^ have been continuously modified and evolved into sonosensitizers with superior performance. Examples include semiconductor-organic conjugated polymers, metal and metal oxide nanoparticles, nanobubbles and acoustically active chemotherapeutic agents.^19^ Nanobiotechnology integrates diagnostics, delivery and therapy into a single nanoplatform. The application of nanobiotechnology presents significant opportunities for the development of nano-sonosensitizers and is expected to maximize the clinical potential of SDT, offering a promising future for cancer diagnosis and treatment.

Some reviews have investigated advancements in sonosensitizers for SDT. Xing *et al.* summarized the recent progress in organic sonosensitizers, highlighting strategies to improve their water solubility, tumor targeting capacity, biocompatibility, and therapeutic effects, as well as the benefits of combining SDT with other cancer treatments.^20^ This review focused on 7 major organic sonosensitizers and SDT-based combination therapies. Qin *et al.* classified inorganic sonosensitizers based on their design principles, emphasizing their mechanisms, chemical properties, and role in tumor microenvironment (TME) regulation, while also exploring TME-engineered nanobiotechnologies and combined therapies to enhance SDT efficacy.^21^ This review main discussed the design principles and TME regulation of inorganic sonosensitizers. Yang *et al.* elaborated on the modification strategies of metal–organic frameworks (MOFs)-based materials and their application as functionalized MOFs-based sonosensitizers in tumor synergistic therapy.^22^ This review specifically examined MOFs-based inorganic sonosensitizers, detailing their structural modifications, material properties, and innovative strategies for addressing various complex conditions. It is worth noting that these reviews primarily focus on either organic or inorganic sonosensitizers, with a strong emphasis on their anti-tumor applications.

Although several recent reviews have comprehensively examined specific classes or aspects of sonosensitizers (*e.g.*, targeted discussions of organic or inorganic materials and their mechanisms), there remains a need for an integrative review that simultaneously (i) systematically categorizes inorganic, organic and inorganic–organic hybrid nano-sonosensitizers, (ii) links these material classes to fundamental SDT mechanisms and design principles, and (iii) critically discusses translational challenges and future clinical pathways. This work is intended to fill that integrative niche by providing a unified perspective bridging materials science, mechanistic insight, and translational considerations. Therefore, this reviews provides an systematic overview of the primary categories of nanobiotechnology-driven nano-sonosensitizers developed in recent years, including inorganic, organic, and inorganic–organic hybrid nano-sonosensitizers, along with other emerging variants. It highlights the latest research advancements and applications of these sonosensitizers. In contrast to previous reviews that mainly focused on either organic or inorganic sonosensitizers and their tumor-related applications,^20,23,24^ this work provides a comprehensive and integrative overview of nanobiotechnology-based SDT systems. It systematically classifies sonosensitizers into inorganic, organic, and inorganic–organic hybrid types, and further includes other emerging variants. More importantly, this review connects the fundamental SDT mechanisms, design strategies, and translational challenges, thus offering a unified perspective that bridges materials science, nanobiotechnology, and clinical medicine. Furthermore, we highlight the role of multifunctional and stimuli-responsive nanoplatforms in advancing SDT toward multimodal precision cancer therapy, which has not been systematically addressed in prior reviews.

## 2. Mechanism and synergistic therapeutic effect of SDT

**Fig. 1:**
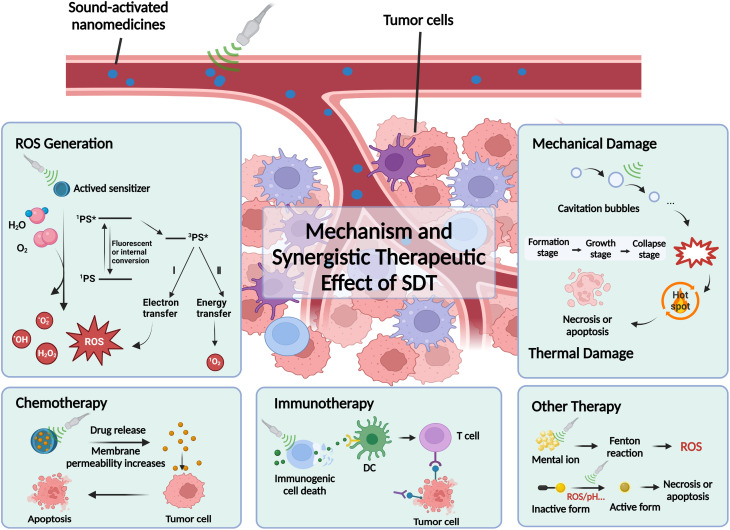
Schematic diagram of the possible mechanisms of SDT and synergistic effect of SDT.

Over the years, the underlying mechanisms of SDT have been extensively investigated. However, due to the complexity of the *in vivo* environment and intricate course of action, no definitive conclusions have been reached. The efficacy of SDT is primarily attributed to the cavitation effect, which occurs when bubbles are formed, expanded, and subsequently collapsed within a liquid under the influence of pressure pulses induced by US.^25^ Cavitation can be classified into two types: inertial cavitation (transient cavitation) and non-inertial cavitation (steady-state cavitation). The ultrasonic cavitation effect has led to the formulation of three main theories: mechanical damage, thermal injury, and the generation of ROS.^26,27^ Among these, ROS generation has been the most extensively studied and widely recognized as a key mechanism underlying SDT therapeutic effects. As shown in Fig. 1, under US irradiation, the sonosensitizer absorbs energy, promoting an electron from the ground state to an excited singlet state. The excited singlet state is highly unstable and can decay back to the ground state through fluorescence or thermal energy dissipation (*i.e.*, acoustic luminescence). In addition, the singlet state can undergo intersystem crossing, where the electron spin converts to a higher energy orbital, resulting in the formation of a more stable and longer-lived excited triplet state. The sonosensitizer in the excited triplet state can then transfer energy directly to molecular oxygen (O_2_), which is referred to as a type II reaction. Alternatively, the excited triplet state may react directly with substrates in the microenvironment—such as cell membranes or biomolecules—undergoing hydrogen or electron transfer to produce radicals and radical ions. This is known as a type I reaction, where the generated radicals further react with O_2_ to produce ROS, including hydroxyl radicals (˙OH), superoxide anions (˙O_2_^−^), and hydrogen peroxide (H_2_O_2_). ROS are known to effectively damage cellular DNA and proteins, promote lipid peroxidation, and induce apoptosis.^25,26,28^ Notably, the generation of ROS is widely considered the primary mechanism underlying the therapeutic effects of SDT.

SDT is often used in combination with other therapies to enhance treatment efficacy, including chemotherapy,^29^ immunotherapy (ICD),^30^ chemodynamic therapy (CDT),^31^ and gas therapy (GT).^32^ As previously mentioned, US plays a dual role in both drug delivery and activation. For drug delivery, US disrupts the structure of drug delivery systems, thereby increasing drug release, while also creating pores in cell membranes to facilitate the transport of therapeutic agents such as small molecules, nucleic acids, and proteins. For drug activation, US stimulates the production of ROS from sonosensitizers, which, in turn, act as effector molecules to induce cell death. Additionally, ROS can activate other synergistic effector molecules, such as gaseous molecules (*e.g.*, carbon monoxide, hydrogen sulfide) or prodrugs containing ROS-sensitive bonds. In conclusion, the combination of SDT with various therapies for the treatment of a broad range of malignant diseases has garnered increasing attention, with encouraging implications for clinical development.^33,34^ Apart from traditional bulk cavitation, recent studies have proposed the theory of intramembrane cavitation, where oscillations of nanobubbles or vapor cavities within the lipid bilayer induce localized mechanical stress, altering membrane integrity and ion transport.^35^ This localized phenomenon may contribute to sonodynamic effects at the subcellular level and facilitate the activation of membrane-associated sonosensitizers.^36,37^ Sonochemical reactions are driven by the extreme microenvironments created during cavitation collapse, including transient temperatures above 5000 K and pressures exceeding several hundred atmospheres.^38^ These conditions generate radicals such as ˙OH, O_2_^−^˙, and singlet oxygen (^1^O_2_), which mediate oxidative stress and cytotoxic effects in SDT. Sonosensitizers serve as catalytic amplifiers in these processes, converting US energy into enhanced ROS yields.^39–41^ From a translational standpoint, nanostructured sonosensitizers that effectively couple with US energy-such as those possessing high acoustic impedance mismatches, piezoelectric components, or electron–hole separation characteristics—demonstrate superior ROS generation efficiency and therapeutic depth. Examples include TiO_2_-based and BaTiO_3_-based systems, as well as conjugated polymer nanoparticles, which offer tunable physicochemical properties for efficient US-energy conversion.^40,42,43^

## 3. Types of sonosensitizers

Sonosensitizers play a crucial role in enhancing the efficacy of SDT, and the advancement of micro/nanotechnology has opened new avenues for the development of more effective and safer sonosensitizers.^44^ Utilizing nano-delivery platforms to encapsulate and deliver small molecule sonosensitizers or developing sound-sensitive nanomaterials, can effectively address the limitations of traditional sonosensitizers and improve the therapeutic outcomes of SDT.^45^ Recently, a variety of sonosensitizers derived from traditional compounds have been extensively studied. Sonosensitizers are primarily classified into three categories: inorganic sonosensitizers, organic sonosensitizers, and inorganic–organic hybrid sonosensitizers.^46^ The following section will provide an overview of the main types and applications of sonosensitizers. This section summarized the new micro/nano sonosensitizers in recent years as shown in Table 1.

**Table 1:** Categorization of inorganic sonosensitizers in recent years

Category	Material	Disease model	US parameters	US time	Ref.
Metal nanomaterials	PtCu NPs	Osteomyelitis	30 kHz, 50% duty cycle	10 min	163
	Pt–VS_4_	Breast cancer	1 MHz, 1.5 W cm^−2^, 50% duty cycle	5 min	136
	BWO-Fe NSs	Breast cancer	1 MHz, 1 W cm^−2^, 50% duty cycle	3 min	66
	MoTe_2_	Breast cancer	1 MHz, 1 W cm^−2^, 50% duty cycle	5 min	59
	Pt–B–P	Ovarian cancer	1 MHz, 2 W cm^−2^	5 min	164
	Cell membrane camouflaged Cu_9_S_8_	Colorectal cancer	1 MHz, 2.5 W cm^−2^, 50% duty cycle	5 min	165
	PCN-224 NP	Glioblastoma	1 MHz, 1 W cm^−2^	4 min	166
	HA@MoCF_3_NPs	Ovarian cancer	1 MHz, 1.5 W cm^−2^	10 min	167
	Fe/Mn-doping-ZnO nanoparticle (D-ZnO NP)	Breast cancer	1 MHz, 1 W cm^−2^, 50% duty cycle	2 min	168
	Ti_2_C(OH)_*X*_	Breast cancer	1 MHz, 1 W cm^−2^, 50% duty cycle	2 min	169
	2D FePS_3_ NS	Breast cancer	1 MHz, 0.5 W cm^−2^, 50% duty cycle	1 min	170
	AuNPs@Ir1	Breast cancer	3 MHz, 0.3 W cm^−2^	20 min	171
	Ag_2_S–Zn_*x*_Cd_1−*x*_S	Breast cancer	1 MHz, 1 W cm^−2^, 50% duty cycle	5 min	54
	LiFePO_4_	Cervical cancer	50 kHz, 3 W cm^−2^, 50% duty cycle	5 min	123
Metal nanomaterials	Au@P–ZnO NRs	Breast cancer	1 MHz, 0.3 W cm^−2^	2 min	172
	MP-Au/ZnO@CCM NRs	Glioma	1 MHz, 1.8 W cm^−2^, 100% duty cycle	2 min	173
	MoO_*X*_	Breast cancer	40 kHz, 3 W cm^−2^	15 min	174
	Ni–CoO@PEG porous NSs	Breast cancer	1 MHz, 1 W cm^−2^, 50% duty cycle	5 min	175
	Au_S/C_–TiO_2_ NSs	Breast cancer	1 MHz, 1.5 W cm^−2^, 50% duty cycle	7 min	56
	2D Pd/H–TiO_2_ NSs	Glioma	1 MHz, 1.5 W cm^−2^, 50% duty cycle	3 min	176
	TiO_2_/C	Pancreatic cancer	1 MHz, 0.5 W cm^−2^, 50% duty cycle	1 min	177
	2D Ti_3_C_2_/CuO_2_@BSA	Glioblastoma	1 MHz, 1 W cm^−2^	1 min	125
	COF@Co_3_O_4_	Breast cancer	50 kHz, 2.0 W cm^−2^	5 min	178
	TiO_2_@g-C_3_N_4_	Breast cancer	1.0 MHz, 1.0 W cm^−2^, 50% duty cycle	5 min	179
	T-mTNPs@L-Arg	Breast cancer	1 MHz, 1 W cm^−2^	1 min	180
	Perovskite-type crystals ZnSnO_3_:Nd	Breast cancer	1 MHz, 0.96 W cm^−2^, 40% duty cycle	2 min	181
Metal nanomaterials	V-v-r BiVO_4_ NSs	Breast cancer	1 MHz, 1.2 W cm^−2^, 50% duty cycle	5 min	182
	(MWO_4_-PEG)NP (M = Fe, Co, Mn, Ni)	Breast cancer	40 kHz, 3 W cm^−2^, 50% duty cycle	5 min	183
	Fe/MnO NPs (FDMN)	Breast cancer	50 40 kHz, 3 W cm^−2^, 50% duty cycle	3 min	184
	CoWO_4_/FeWO_4_ heterojunction	Breast cancer	1 MHz, 0.5 W cm^−2^	10 min	185
	CoFe_2_O_4_	Breast cancer	1 MHz, 0.5 W cm^−2^, 20% duty cycle	3 min	186
	WO_*x*_NBs	Breast cancer	50 kHz, 3 W cm^−2^, 2 cycles (5 min each cycle)	5 min	187
	CuS/TiO_2_	Glioblastoma	0.5 W cm^−2^	10 min	166
	Cu_2−*x*_O@TiO_2−*y*_ heterojunction	Human osteosarcoma	50 kHz, 2 W cm^−2^	5 min	188
	H_*X*_V_2_O_5_ (HVO)	Breast cancer	40 kHz, 3 W cm^−2^	5 min	189
	MO@FHO	Breast cancer	1 MHz, 1.5 W cm^−2^	2 min	190
Nonmetallic semiconductors	C-60 fullerene	Cervical cancer	1 MHz, 5.4 W cm^−2^	1 min	191
Nonmetallic semiconductors	2D stanene-based nanosheets	Lung cancer	1 MHz, 1 W cm^−2^, 50% duty cycle	1 min on and 1 min off for 3 on/off cycles	75
	C_60_–s-BP	Breast cancer	1 MHz, 1 W cm^−2^	5 min	192 and 193
	CD@Ti_3_C_2_T_*x*_ HJs heterojunction	Breast cancer	50 kHz, 3 W cm^−2^	5 min	194
	Pt/N-CD@TiO_2−*x*_	Human osteosarcoma	50 kHz, 2 W cm^−2^	5 min	195

### 3.1 Inorganic sonosensitizers

Inorganic nanomaterials have gained widespread use as biosensitizers in the biomedical field due to their high stability and superior physicochemical properties.^47^ First, the structural integrity of inorganic nanomaterials remains intact under US irradiation, making them highly stable during SDT.^48^ Second, these materials exhibit minimal toxicity in the absence of the US, which contributes to their safety for therapeutic applications. Furthermore, inorganic nanomaterials can be easily functionalized or combined with other materials to enhance or expand their therapeutic properties.^49^ Given these advantages, the development and application of inorganic sonosensitizers are crucial for improving the efficacy and safety of SDT.

#### 3.1.1 Metal nanoparticles

**Fig. 2:**
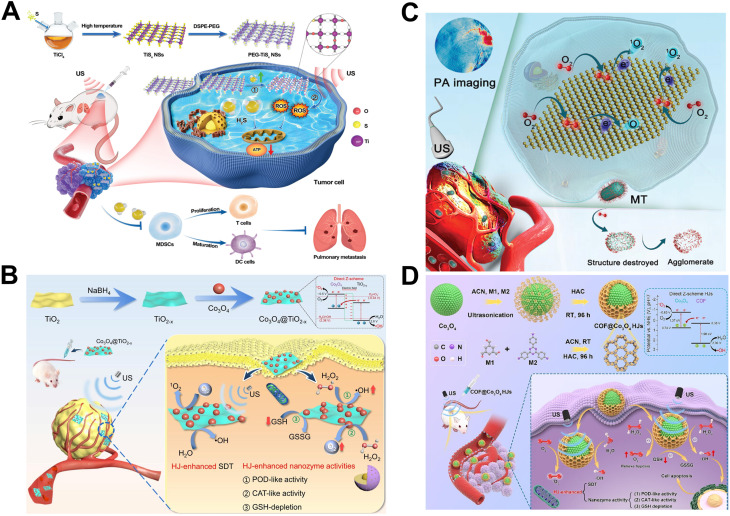
(A) Illustration of the preparation method for titanium sulfide nanosheets and a schematic representation of the synergistic cancer treatment combining SDT and GT. (B) Co_3_O_4_@TiO_2−*x*_ HJs nanomaterials integrate SDT with nanocatalytic therapy to modulate the TME, enhancing ROS production by decreasing GSH level at the tumor site and alleviating hypoxia *via* triple mimetic catalytic activity. (C) Molybdenum telluride facilitates photoacoustic imaging guided SDT. (D) Schematic of COF@Co_3_O_4_ HJs synthesis and diagram showing improved carrier separation and enhanced sound sensitivity. (A) Reproduced with permission.^57^ Copyright 2022, John Wiley & Sons. (B) Reproduced with permission.^58^ (C) Reproduced with permission.^59^ Copyright 2022, John Wiley & Sons. (D) Reproduced with permission.^60^ Copyright 2023, Elsevier.

Among metal nanoparticles, transition metals (*e.g.*, titanium, platinum, copper and rhodium and their oxides) as well as precious metal nanomaterials (*e.g.*, gold and silver) are widely recognized as sonosensitive materials due to their high stability and enhanced ultrasonic contrast. These metal nanoparticles often exhibit multiple enzyme-like catalytic activities, such as oxidase-like activity, catalase-like activity, earning them the designation of nanoenzymes.^50,51^ TiO_2_, a common inorganic semiconductor material, is extensively utilized in fields of energy and food biomedicine.^52^ Numerous studies demonstrated that TiO_2_ can generate ROS and induce apoptosis in tumor cells when activated by the US, positioning it as a promising sonosensitizers.^53^ However, pure TiO_2_ suffers from several limitations, including aggregation, facile electrons–holes recombination, and restricted functionality. As a result, researchers have sought to overcome these challenges through various modifications to enhance its SDT performance.^54^ To address the issue of easy aggregation, You *et al.* modified the surface of TiO_2_ with carboxymethyl dextran, which improved its stability and prolonged circulation time, thereby enhancing tumor accumulation.^55^ To overcome the challenge electron–hole recombination, Lu *et al.* employed metal ion doping method to promote electron–hole separation and increase the quantum yield. Specifically, they dispersed gold atom (Au) onto the surface of TiO_2_ nanosheets (denoted as Au_S/C_–TiO_2_). The Au atoms and Au clusters deposited on TiO_2_ nanosheets effectively enhanced the separation of electron–hole pairs. Furthermore, the incorporation of Au imparted glucose oxidase-like catalytic activity to the sonosensitizers. *In vivo* experiments involving immune cell demonstrated that Au_S/C_–TiO_2_ not only induced apoptosis in tumor cells but also promoted the generation of tumor immune cells, highlighting its potential for tumor treatment.^56^ To enhance the functional diversity of TiO_2_, researchers have developed a series of multifunctional sonosensitizers that enabled the combined application of multiple therapies. As shown in Fig. 2, Li *et al.* developed titanium sulfide nanosheets (TiS_*X*_NSs), which can autonomously release H_2_S gas *in situ*. The high concentration of H_2_S inhibits cytochrome C oxidase, disrupting the mitochondrial electron transport chain. As H_2_S is release, sulfur vacancies form on the TiS_*X*_NSs surface, leading to the generation of TiO_*X*_, which further enhances the therapeutic efficacy. Importantly, the metabolizable TiS_*X*_NSs were efficiently eliminated from the body without causing significant long-term toxicity. This study expanded the biological applications of TMD-based nanoplatforms, offering valuable insights for future research, as shown in Fig. 2A.^57^ Similarly, the therapeutic efficacy of SDT was enhanced through the integration of nanocatalysis therapy, which remodeled the TME to achieve cascade amplification of ROS. The Co_3_O_4_@TiO_2_ were developed based on heterojunction (HJ) enhancement and exhibited triple enzyme-like catalytic activities, including peroxidase-like catalytic activity, catalase-like catalytic activity and glutathione (GSH) depletion catalytic activity. These Co_3_O_4_@TiO_2−*x*_ HJs demonstrated superior catalytic activities compared to single Co_3_O_4_ NPs and TiO_2−*x*_ NSs. They effectively regulated the TME by reducing the GSH level, alleviating tumor hypoxia and enhancing ROS generation, ultimately leading to the complete eradication of the tumor, as shown in Fig. 2B.^58^

In addition to TiO_2_ nanoparticles, molybdenum ditelluride nanomaterials exhibited excellent biocompatibility, biodegradability and remarkable catalytic activity, enabling photoacoustic-guided SDT and enhancing their clinical applicability, as shown in Fig. 2C.^59^ Moreover, the Z-scheme heterojunction COF@Co_3_O_4_ with a core–shell structure enhanced sonosensitivity by improving carrier separation dynamics, thereby boosting its multiple enzyme-like catalytic activities. This approach represents a promising strategy for designing semiconductor heterojunction materials with diverse enzyme-like catalytic and sonodynamic properties, as shown in Fig. 2D.^60^ However, a potential concern with metal nanomaterials is the alteration of the valence state of metal ions during catalysis, which could disrupt the body's regulatory systems and lead to imbalances in the internal environment. Therefore, the exploration of metal catalytic materials with stable, invariant valence states is crucial for improving biological safety. Fu *et al.* discovered that CaF_2_ exhibited peroxidase-like activity, oxidize 3,3′,5,5′-tetramethylbenzidine without changing its valence state.^61^ This groundbreaking finding prompted further research into the biomedical applications of CaF_2_. Subsequently, Dong *et al.* developed CaF_2_ nanozymes, which demonstrated that the Ca^2+^ overload generated during catalysis could decrease mitochondrial membrane potential and ATP levels, ultimately disrupting cellular homeostasis and inducing apoptosis.^62^ This mechanism improved the efficacy of nanocatalytic therapy for tumors. The discovery of valence-invariant metallocene nanocrystals has paved the way for the development of biologically safe nanoenzymatic materials, offering a promising direction for future research.

#### 3.1.2 Piezoelectric materials

The primary factor limiting the generation of ROS in sonosensitizers is the rapid recombination of electron–hole. Therefore, enhancing charge separation and preventing electron–hole recombination are crucial for optimizing the performance of sonosensitizers. Piezoelectric semiconductor materials have demonstrated the ability to effectively promote electron–hole separation and inhibit recombination due to the piezoelectric effect, thereby efficient generation of ROS. Many metals, including metals such as BaTiO_3_, MoS_2_ and ZnO, exhibit piezoelectric properties.^63,64^ Moreover, researchers have sought to optimize traditional piezoelectric materials to further enhance their performance. Studies have shown that metal doping and the creation of crystal defects can significantly improve the piezoelectric effect. These modifications are believed to work by introducing electron traps, which help prevent electron–hole recombination, thereby enhancing the overall efficiency of ROS generation in sonosensitizers.^65^ In recent years, doped piezoelectric materials, particularly those incorporated with metals, have attracted significant attention. For example, iron was introduced into bismuth tungstate piezoelectric material to form Fenton-active nanosheets (BWO-Fe NSs). As the iron doping concentration increased, the amount of ˙OH generated *via* the Fenton reaction increased, leading to enhanced SDT. Both *in vivo* and *in vitro* studies demonstrated that BWO-Fe NSs-mediated SDT effectively inhibited the growth of refractory breast cancer in mice.^66^ Similarly, Cheng *et al.* incorporated bismuth ions into barium titanate, while Liu *et al.* developed a gadolinium-doped zinc oxide sonosensitizer for tumor SDT eradication. These studies aim to explore the impact of metals doping on improving charge separation, ultimately enhancing the sonosensitivity of piezoelectric materials for more efficient SDT. The findings confirmed that this approach is indeed an effective method for achieving the desired outcomes.^67^ Metal doping can significantly enhance the catalytic activity to sonosensitizers. For example, Zhao *et al.* developed MnO_*x*_-modified BiOCl nanosheets, where the incorporation of MnO_*x*_ not only endowed the nanosheets with catalyst-like properties, enabling them to decompose intracellular H_2_O_2_ to generated O_2_ and alleviated tumor hypoxia, but also enhanced the piezoelectric effect under the US, further improving the efficiency of ROS generation.^63^ However, overcoming the band gap barrier to directly generate charges *via* high piezoelectric voltage remained a challenge. Building on previous research, Zhu *et al.* constructed Mn–Ti bimetallic organic framework square nanosheets. These nanosheets were shown to achieve sonopiezocatalytic dynamic therapy through high piezoelectric voltage generation, exhibiting significant antitumor efficacy both *in vivo* and *in vitro*.^68^

In addition to the piezoelectric effect, good biocompatibility and biodegradability are essential characteristics for successful *in vivo* applications of sonosensitizers. Chen *et al.* developed core–shell structured nanoparticles based on traditional piezoelectric material BaTiO_3_ (BTO–Pd–MnO_2_–HA). The hyaluronic acid (HA) coating on the surface enhanced both the targeting and stability of the sonosensitizers. The MnO_2_ shell exhibited enzyme-like catalytic activity, reacting with H_2_O_2_ to produce O_2_, which alleviated tumor hypoxia and further activated the piezoelectric material to generate additional ROS. Studies demonstrated that BTO–Pd–MnO_2_–HA nanoparticles significantly enhanced ROS production, marking the first development of degradable core–shell type piezoelectric nanomaterials. This research expands the potential application of piezoelectric nanomaterials in combination with US-triggered cascade amplification therapies in biomedical fields.^69^

#### 3.1.3 Nonmetallic semiconductors

**Fig. 3:**
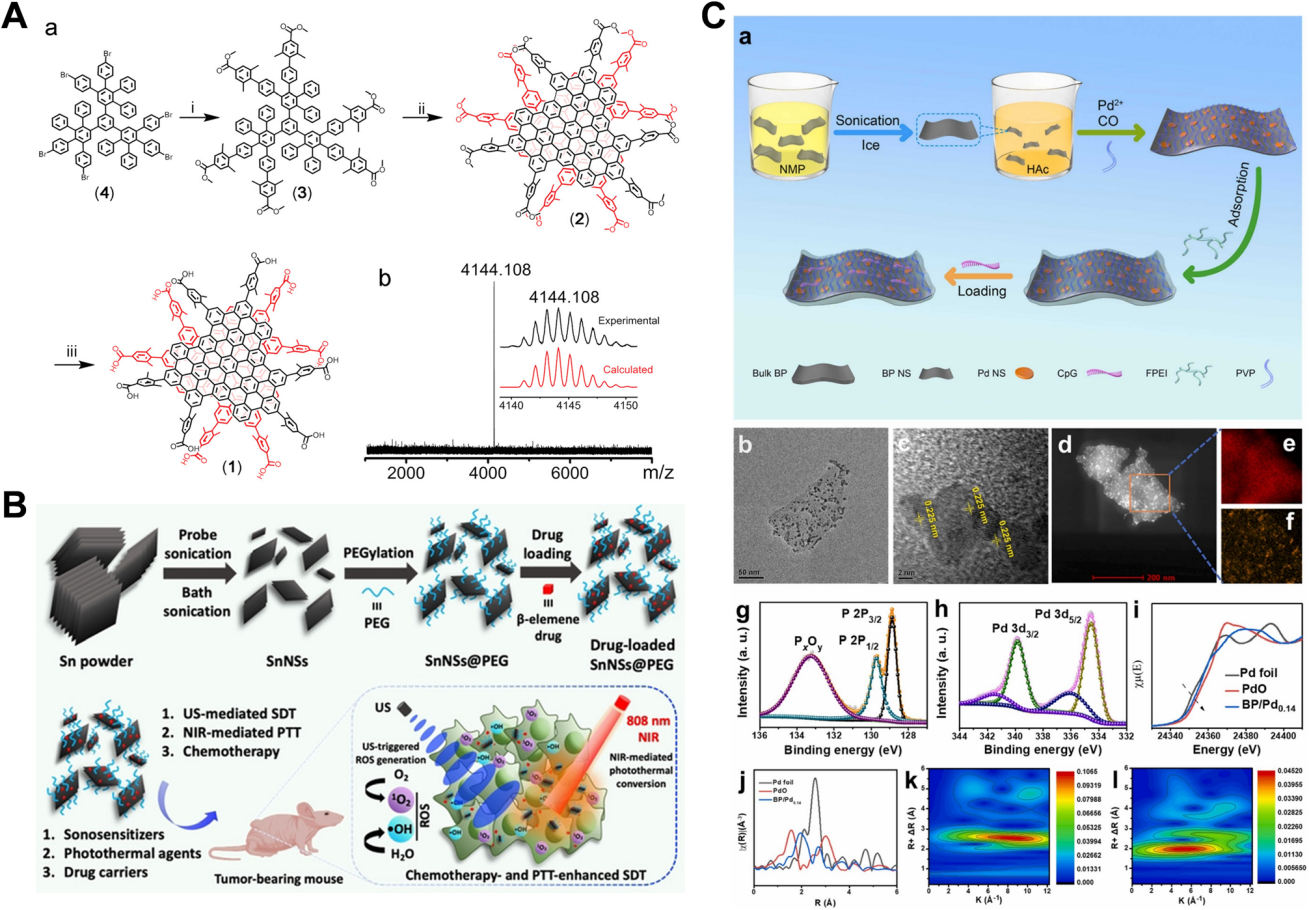
(A) Diagram showing the synthesis process of WAGQD-C_96_. (B) Schematic representation of the preparation of polyethylene glycol (PEG)-functionalized 2D stanene nanosheets (SnNSs@PEG) using a liquid-phase exfoliation technique, along with their application in chemotherapy and PTT-enhanced SDT. (C) Overview of the synthesis process and structural characterization. (A) Reproduced with permission.^73^ Copyright 2022, John Wiley & Sons. (B) Reproduced with permission.^75^ Copyright 2021, John Wiley & Sons. (C) Reproduced with permission.^80^ Copyright 2022, Elsevier.

In addition to the metallic semiconductor materials previously discussed, certain non-metallic materials also exhibit semiconductor properties, including carbon-based materials and black phosphorus.^70^ Carbon-based materials, in particular, possess excellent semiconductor characteristics. Compared to metal nanoparticles, carbon-based nanomaterials offer a larger surface area and higher conductivity, which effectively facilitates the separation of electron–hole pairs. Materials such as graphene, carbon nanotubes and fullerenes have significant attention in research.^71^ For example, graphene (GR) and its derivatives are widely used in biosensing, drug delivery and cancer treatment due to their large specific surface area, tunable structure and good biocompatibility. Numerous intelligent response platforms based on GR and its derivatives are currently employed for SDT and combination therapies for cancer.^72^ However, many natural graphene materials exhibit strong π–π interactions, resulting in poor water solubility, which limits their applications in the biomedical field. To overcome this challenge, the researchers did a lot of exploring as shown in Fig. 3, Shi *et al.* developed water-soluble WAGQD-C_96_, which demonstrated superior sonodynamic properties and could be effectively taken up by cells and retained in tumor tissues, as shown in Fig. 3A.^73^ Two-dimensional stannene (IVA), a derivatives of GR, has attracted substantial research interest due to its unique electronic structures, exceptional quantum effects and potential superconductivity.^74^ For instance, Chen *et al.* developed IVA nanosheets as sonosensitizers and drug carriers for PDT, PTT and SDT-mediated cancer treatments. This work not only provided an effective method for large-scale synthesis of nanosheets but also established a universal platform for tri-modal combination cancer therapies, as shown in Fig. 3B.^75^

Black phosphorus (BP) is another prominent non-metallic semiconductor material, notable for its layered structure, which provides a large specific surface area and multiple nucleation sites, making it highly promising for SDT.^76^ The use of BP nanosheets (BP NSs) as sonosensitizers was first reported in 2015, marking a significant advancement in the field.^77^ Since then, research on BP-based sonosensitizers has expanded considerably. It has been demonstrated that the toxicity of BP is dose-dependent, underscoring the importance of carefully controlling the dosage to ensure the safety of BP as a sonosensitizer.^78,79^ Additionally, in the context of combination therapies, Fu *et al.* pioneered a “three-in-one” multi-modal strategy, developing the two-dimensional (2D) nanoplatform based on black phosphorus/palladium (BP/Pd–FPEI–CpG) for synergistic tumor PDT/SDT/CDT. This approach not only provided insights into multi-modal therapy but also contributed to advancements in multi-modality imaging, showcasing the potential of BP in enhancing therapeutic outcomes across different treatment modalities, as shown in Fig. 3C.^80^

Phosphorescent materials, particularly those emitting near-infrared (NIR) range, are widely used as diagnostic agents for NIR imaging due to their ultra-long carrier lifetimes and narrow bandgaps. These materials have also evolved into promising sonosensitizers for image-guided SDT. However, most conventional NIR phosphorescent materials are based on transition metal complexes (Re, Ru, Os, IR, Pt, Pd and Au), which are associated with high metal toxicity and cost, limiting their clinical applications.^81,82^ Therefore, the development of non-metallic NIR phosphorescent materials with good biocompatibility and low cost represents a potential breakthrough for advancing image-guided SDT. Recent have focused on designing NIR-emitting sonosensitizers using carbon dot materials, which featured narrow bandgaps and long-lived excited triplet states. NIR phosphorescent carbon dots have shown enhanced SDT efficiency under low-intensity US irradiation, achieving complete eradication of solid tumors with a single injection and irradiation. This research opened up exciting possibilities for the development of phosphorescent materials with long-lived triplet excited states, offering a promising strategy for precise and effective tumor treatment.^83^

### 3.2 Organic sonosensitizers

Early studies of SDT, identified natural organic small molecules, such as porphyrins and dihydroxybipyrroles, as excellent sonosensitizers due to their strong acoustic activity. These compounds have since become the focus of extensive research in the biomedical field.^84^ The integration of traditional organic small-molecule sonosensitizers with nanotechnology can enhance their physicochemical properties, improving drug delivery targeting, responsiveness to the tumor microenvironment, and enabling combination therapies.^85^ Moreover, rational structural optimisation and the design of nano-drug based on traditional organic sonosensitizers hold the potential to maximize their intrinsic value, facilitating the clinical translation of organic sonosensitizers (Table 2).

**Table 2:** Categorization of organic sonosensitizers in recent years

Category	Material	Disease model	US parameters	US time	Ref.
Organic small molecule-based sensitizers	HAL/3MA@X-MP	Breast cancer	1 MHz, 1.4 W cm^−2^	1 min	88
	Prodrug of tetrahydroxyporphyrin	Breast cancer	0.85 MHz, 0.03 W cm^−2^, 50% duty cycle	10 min	196
	H-Pys-HA@M/R	Breast cancer	1 MHz, 1.6 W cm^−2^, 50% duty cycle 0.85 MHz, 0.03 W cm^−2^, 50% duty cycle	5 min	197
	GdPorP	Liver neoplasms	1 MHz, 1.5 W cm^−2^, 50% duty cycle	1 min	198
	pPC-TK	Liver cancer	1 MHz, 0.5 W cm^−2^	10 min	91
	FeS_2_@PcD	Cervical cancer	1 MHz, 1 W cm^−2^	5 min	199
	BTeTh-NPs	Breast cancer	1 MHz, 1 W cm^−2^	3 min	170 and 200
	Aza-BDY NPs	Breast cancer	1 W cm^−2^	10 min	172
	OA/Ce6	Breast cancer	1 MHz, 0.1 W cm^−2^, 100% duty cycle	90 s	201
	B-OH@Cy5-PEG-NH_2_ NSs	Breast cancer	1 MHz, 1 W cm^−2^, 50% duty cycle	5 min	202
	CAT@SiO_2_-ICG@Ex-A	Glioblastoma	1 MHz, 1.5 W cm^−2^, 40% duty cycle	5 min	203
	TPGS–PEM–ICG	Cervical cancer	1 MHz, 1 W cm^−2^	5 min	204
Organic small molecule-based sensitizers	ICG/OXP	Ovarian cancer	1 W cm^−2^	1 min	205
	M1/IR780@PLGA NPs	Breast cancer	1 MHz, 2.0 W cm^−2^	30 s on and 30 s off for 4 on/off cycles	97
	IR780/RSL-3 PLGANPs	Breast cancer	3 W cm^−2^	2 min on and 2 min off for 2 on/off cycles	206
	IR780/PTX-TL-PEG_1K_-NH_2_-NPs	Glioma	1 MHz, 0.2∼0.4 W cm^−2^	3 min	96
	IR780/melbine lipidosome	Breast cancer	1 MHz, 1 W cm^−2^	1 min	207
	Gox/HMME/IR780 PLGA NPs	Breast cancer	2 W cm^−2^	5 min	208
Semiconductive polymers	Semiconductor conjugated polymer coupling MSA-2	Squamous cell carcinoma	50 kHz, 1 W cm^−2^, 50% duty cycle	6 min	209
	SPN_Ab_	Breast cancer	1 MHz, 1.5 W cm^−2^, 50% duty cycle	6 min	102
Semiconductive polymers	SPN/catalase	Breast cancer	50 kHz, 1 W cm^−2^, 50% duty cycle	5 min	104
	PEG-*b*-IR/sabutoclax/Mn^2+^/PEG-*b*-Pho	Breast cancer	1 MHz, 2.5 W cm^−2^, 50% duty cycle	5 min	210

#### 3.2.1 Organic small molecule-based sonosensitizers

**Fig. 4:**
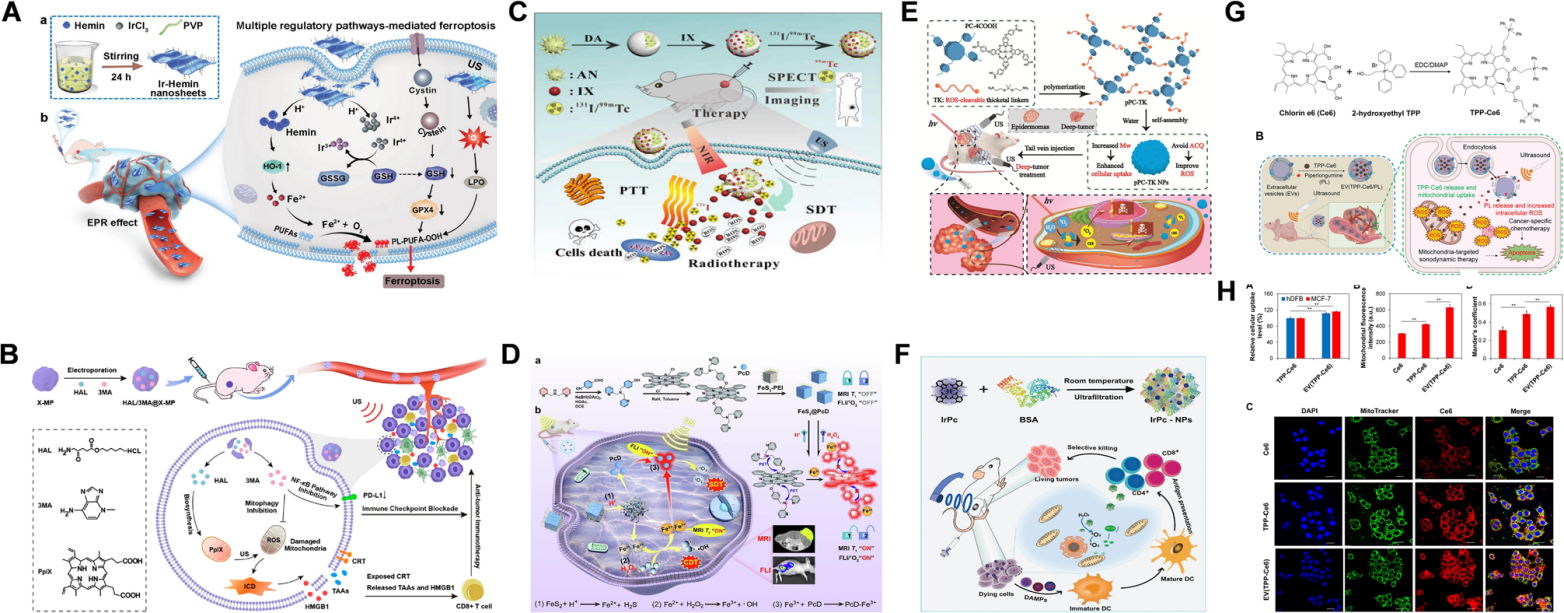
(A) Diagram illustrating iridium-coordinated nanosheets designed to induce tumor ferroptosis through multiple regulatory mechanisms. (B) Schematic representation of the preparation of HAL/3MA@X-MP and its antitumor effects. (C) Overview of the synthesis pathway for ^131^I/^99m^Tc-AN@D/IX and its combined radionuclide, sonodynamic, and photothermal therapies. (D) Fabrication process and programmed mechanism of FeS_2_@PcD, as well as its action in HepG2 hepatocarcinoma cells, enabling fluorescence/MR dual-modality imaging-guided sono/chemodynamic therapies. (E) Schematic diagram of the preparation of pPC-TK NPs and their *in vivo* anticancer effects *via* PDT, GSH and SDT. (F) Synthetic route for IrPc-NPs and their role in sonodynamic immunotherapy. (G) Chemical synthesis of TPP-Ce6 and the schematic representation of combined chemo-SDT using biocompatible EVs encapsulating mitochondria-targeting TPP-Ce6 and the pro-oxidant piperlongumine (PL). (H) Enhanced cellular uptake of drug-loaded EVs, with EV(TPP-Ce6) showing significantly higher internalization in both hDFB and MCF-7 cells compared to free TPP-Ce6. (A) Reproduced with permission.^87^ Copyright 2023, John Wiley & Sons. (B) Reproduced with permission.^88^ Copyright 2023, American Chemical Society. (C) Reproduced with permission.^66^ Copyright 2023, Elsevier. (D) Reproduced with permission.^93^ Copyright 2023, Elsevier. (E) Reproduced with permission.^91^ Copyright 2023, John Wiley & Sons. (F) Reproduced with permission.^94^ Copyright 2022, John Wiley & Sons. (G and H) reproduced with permission.^89^ Copyright 2023, Elsevier.

Porphyrins and their derivatives are a class of natural organic molecules with aromatic macrocyclic structures, widely found in organisms and crucial cellular organelles involved in energy transfer.^86^ In animals, they are primarily present in heme (iron porphyrin) and hemocyanin (copper porphyrin). These molecules have been used in disease treatment and biomedical imaging since the last century.^87^ Porphyrins are capable of inducing ROS production under US irradiation, which can damage cell membranes, mitochondria, proteins, and DNA. Currently, porphyrins are clinically approved for treating lung cancer, skin cancer and oesophagal cancer^8^ However, free porphyrins face limitations, including poor water solubility and rapid elimination from the bloodstream. To address these challenges, researchers have developed a range of porphyrin-based sonosensitizers using nanomedicine design strategies. For example, as shown in Fig. 4, iridium-coordinated hemin nanoparticles were synthesized. Under US irradiation, these nanoparticles targeted and accumulated at tumor sites, where they rapidly degrade to release hemin and Ir. This degradation process depleted intracellular GSH, leading to the inactivation of glutathione peroxidase 4 and thereby synergistically amplifying ferroptosis in tumor cells, as shown in Fig. 4A.^87^ In another study, An *et al.* developed dopamine-modified Au nanostars as carriers, labelled with diagnostic radionuclides (^131^I or ^99m^Tc) and loaded with protoporphyrin IX, forming a radiolabeled nano delivery system ^131^I/^99m^Tc-AN@D/IX. Single-photon emission computed tomography imaging showed that, after administration, a significant portion of the nanoparticles remained localized within tumors, enhancing sensitizer-mediated SDT. This study represented the first demonstration of combined radioisotope therapy, SDT and photothermal therapy (RNT–SDT–PTT), offering promising prospects for effective treatment of pancreatic tumor, as shown in Fig. 4C.^66^ To improve the safety of sensitizers, Zuo *et al.* developed a dual-responsive sonosensitisers that responds to both GSH and US. This was achieved by designing a prodrug, 2,4-dinitrobenzene sulfonyl, which was conjugated to tetrahydroxyporphyrin and self-assembled into nanoparticles with DSPE-PEG. The coupling of the quencher significantly inhibited the acoustic activity of the tetrahydroxyporphyrin, which was not activated by US irradiation alone. Instead, activation occurred only in the presence of elevated levels of GSH, triggering strong fluorescence emission and ROS production. This dual ROS/GSH responsive mechanism significantly enhanced biological safety of the therapy while reducing the side effects typically associated with cancer treatments, as shown in Fig. 4.^88^ Chlorin e6 (Ce6), a derivative of porphyrin, tends to aggregate in solution due to its high hydrophobicity and often requires modification or combined application with steroids, proteins, phospholipids, or inorganic nanomaterials to enhance its efficacy. For example, Ce6 was covalently linked to a cyclometalated iridium complex, forming a conjugate that specifically targets and localizes in the mitochondria, making it suitable for deep-seated tumor therapy under US and two-photon excitation, as shown in Fig. 4G and H.^89^ Similarly, Cao *et al.* developed extracellular vesicles (EVs) co-loaded with triphenylphosphonium-modified Ce6 (TPP-Ce6), which enhanced cellular uptake and mitochondrial targeting. *In vivo* anti-cancer studies in MCF-7 tumor-bearing mice showed that EV(TPP-Ce6/PL) combined with US treatment significantly inhibited tumor growth without inducing systemic toxicity, highlighting the potential of improving the biocompatibility of sonosensitizers.^90^

Phthalocyanine, an organic molecules structurally and functionally analogous to porphyrins, is a fully synthetic compound. Compared to porphyrins, phthalocyanine exhibits a higher triplet quantum yield, a longer triplet state lifetime, reduced absorption of natural light and lower toxicity. Its structure allows for easy modification to improve physicochemical properties, meeting diverse therapeutic requirements. For example, the carboxyl group of phthalocyanine was conjugated with an amino group *via* an active oxygen cleavable thio-ketone linker to form a polymer that self-assemble into nanoparticles approximately 48 nm in size. These nanoparticles demonstrated superior efficacy in inhibiting deep-seated liver tumor growth compared to monomeric phthalocyanines, as shown in Fig. 4E.^91^ As with porphyrins, metal coordination significantly enhances the activity of phthalocyanines, resulting in metal phthalocyanines—compounds that structurally resemble iron porphyrins in hemoglobin and exhibit considerable acoustic activity. Metal phthalocyanines have been extensively studied for biomedical applications.^92^ Notable examples include iridium phthalocyanine nanoparticles (IrPcs NPs), which exhibit high biocompatibility and used in sono/immunotherapy dynamic therapy, pH and H_2_O_2_ dual-responsive phthalocyanine-iron-based nano-reactors (FeS_2_@PcD), as shown in Fig. 4D,^93^ which enable fluorescence/magnetic resonance dual-modal imaging-guided sono/chemical dynamic therapy. These advancements underscore the promising potential of metal phthalocyanines in the biomedical field.

**Fig. 5:**
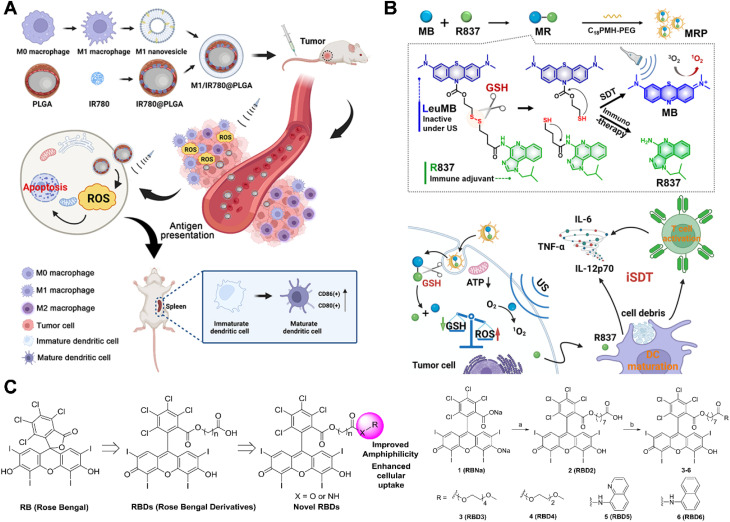
(A) Diagram depicting the M1/IR780@PLGA nanoparticles for antitumor sonodynamic therapy (SDT) and their role in converting M2 tumor-associated macrophages into the M1 phenotype, stimulating dendritic cell maturation, and inducing a robust antitumor immune response. (B) Schematic representation of MB-R837-PEG (MRP) nanoparticles designed for GSH-activated immunosonodynamic therapy. (C) Chemical structures of RB and its newly developed derivatives. (A) Reproduced with permission.^97^ Copyright 2022, Dove Medical Press Ltd. (B) Reproduced with permission.^98^ Copyright 2022, American Chemical Society. (C) Reproduced with permission.^100^ Copyright 2018, Elsevier.

Indole-based cyanine, particularly IR780, have been extensively studied as sonosensitizers. For example, Ma *et al.* developed IR780@O_2_-SFNs/iRGD as an oxygen-sufficient and tumor-penetrating nanoplatform that overcomes oxygen supply limitations and restricted tumor penetration in PDT, significantly inhibiting primary tumor growth and reducing lung and liver metastases.^95^ Wu *et al.* developed nanoparticles (IR780/PTL-NPs) loaded with IR780 and a paclitaxel prodrug (PTX-TL), linked *via* a ROS-sensitive thioketal linker (TL). The results showed that under US irradiation, IR780 generated ROS, which cleaved the TL to release PTX and effectively inhibiting tumor growth in glioma-bearing mice without significant toxicity.^96^ In another study, Chen *et al.* designed macrophage-derived nanoparticles (M1/IR780@PLGA) by coating IR780-loaded poly(lactic-*co*-glycolic acid) (PLGA) nanoparticles with macrophage membranes. The presence of these membranes enhance homologous targeting, promoting dendritic cells maturation in the tumor microenvironment and repolarized M1 macrophages. This strategy effectively prevented premature drug leakage, significantly improving the biocompatibility and safety of the therapeutic system. The combination of SDT and ICD further enhanced tumor therapeutic efficacy, demonstrating the promising clinical potential of bio-nanosystems for targeted therapy, as shown in Fig. 5A.^97^

Phenothiazine compounds, characterized by polycyclic structures, include methylene blue (MB), promethazine hydrochloride, and prochlorperazine hydrochloride. MB is widely utilized in chemical indicators, dyes and pharmaceuticals. In a recent study, researchers developed self-assembled nanoparticles (MRP NPs) by linking a GSH-sensitive, disulfide bond-reduced form of MB (Leu-MB) with toll-like receptor agonist imiquimod (R837). When exposed to the TME, Leu-MB underwent redox reactions to activate MB, which, under US induction, exhibited cytotoxicity. In parallel, R837 triggered the host ICD response, thereby facilitating immune SDT, as shown in Fig. 5B.^98^

Furthermore, fluorescent dyes, such as rose bengal (RB) and erythromycin B (EB), have been shown to exhibit acoustic activity, with their cytotoxicity significantly enhanced under US irradiation. However, the physicochemical properties of these dyes pose a major limitation to their potential clinical application. For example, RB high hydrophilicity and low tumor accumulation hinder its efficacy *in vivo*.^99^ To address this issue, chemical modification of RB is necessary. In a study by Chen *et al.* RB was chemically modified with methoxy polyethylene glycol, 8-aminoquinoline and 1-naphthylamine to create a series of rosmarinic derivatives (RBDs). These derivatives exhibited strong fluorescence absorption at 548 nm compared with RBs. Additionally, cellular experiments demonstrated that the RBDs exhibited higher cellular uptake and more effective growth inhibition rates. Notably, the RB derivatives modified with methoxy polyethylene glycol (RBD4)-mediated SDT demonstrated the most significant anti-tumor effects when used in SDT, as shown in Fig. 5C.^100^

#### 3.2.2 Semiconductor polymer-based sonosensitizers

Semiconductor polymers are a class of large organic molecules characterized by carbon-based main chains and extended conjugated structures. When these polymers form nanoparticles, they are referred to as semiconductor polymer nanoparticles (SPNs). Research has demonstrated several advantages of semiconductor polymers and their derivatives, including tunable fluorescence emission wavelengths, flexible chemical structures, good stability and biocompatibility. Due to these properties, they are increasingly studied as imaging agents, photothermal therapy agents and sonosensitizers,^101^ particularly in the context of SDT.^14^

**Fig. 6:**
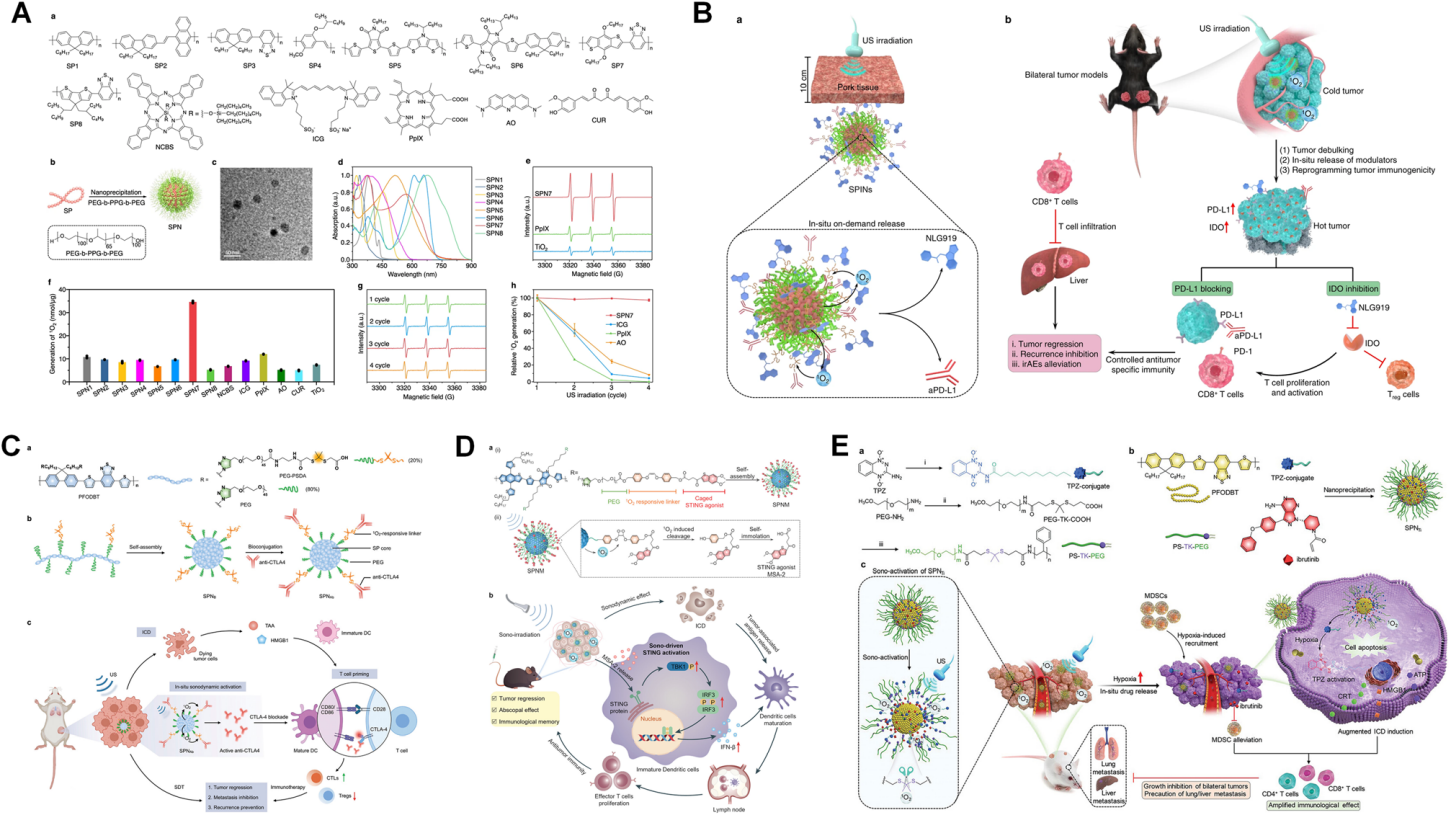
(A) Overview of SPNs screening for SDT. (B) Concept and mechanism of SPINs for deep-tissue activatable sono-immunotherapy. (C) Diagram illustrating SPNAb-mediated activatable sono-immunotherapy. (a) Chemical structure of PFODBT-PEG-PSDA. (b) Step-by-step preparation of SPNAb. (c) Mechanism of SPNAb-mediated sono-immunotherapy, highlighting TAA (tumor-associated antigen), DC (dendritic cell), HMGB1 (high mobility group box 1 protein), CTL (cytotoxic T lymphocyte), and Treg (regulatory T cell). (D) Illustration of SPNM-mediated sono-driven STING pathway activation for sono-immunotherapy in HNSCC. (a) Chemical structure and synthesis of SPNM, with sonodynamic cleavage of the diphenoxyethene bond releasing MSA-2 from SPNM. (b) Proposed mechanism of SPNM-mediated sonodynamic effect, involving STING activation, ICD induction, cytokine secretion, dendritic cell maturation, and effector T cell proliferation to enhance antitumor immunity. (E) Design of sono-activatable SPNTi for improved immunotherapy. (A and B) Reproduced with permission.^103^ Copyright 2023, Nature Publishing Group. (C) Reproduced with permission.^102^ Copyright 2022, John Wiley & Sons. (D) Reproduced with permission.^106^ Copyright 2023, John Wiley & Sons. (E) Reproduced with permission.^107^ Copyright 2023, John Wiley & Sons.

Semiconductor polymers offer flexible and adaptable structures, making them ideal for dual functions as drug delivery vehicles and sonosensitizers.^102^ Recent studies have employed chemical modifications to develop a series of US-responsive compounds based on semiconductor polymers. For example, Li *et al.* conjugated two immune modulators, NLG919 and aPD-L1, sequentially *via*^1^O_2_-cleavable linkers onto the surface of semiconductor polymers, creating semiconductor immune modulating nanoparticles (SPINs). These SPINs effectively accumulated in subcutaneous pancreatic tumors in mice and orthotopic pancreatic tumors in rabbits. Upon US irradiation, they generated ^1^O_2_, which triggered the release immune modulators. This process upregulated the expression of IDO and PD-L1, reshaping the tumor microenvironment by converting non-immunogenic “cold” tumors into immunogenic “hot” tumors. This activated of ICD and effectively eliminated pancreatic tumors in mice while preventing recurrence, as shown in Fig. 6A and B.^103^ Similarly, in another study, Zeng *et al.* used a ^1^O_2_-cleavable linkers to conjugate anti-CTLA-4 antibodies onto polymer nanoparticles (SPN_Ab_). Under US irradiation, these SPN_Ab_ were activated, generating ^1^O_2_, which promoted the release of the anti-CTLA-4 antibody and induced ICD. This process enhanced the proliferation of cytotoxic T-lymphocytes, reduced immunosuppressive regulatory T-cells and contributed to tumor regression, inhibition of metastasis, prevention of recurrence and the establishment of a long-lasting immune memory, as shown in Fig. 6C.^102^ Additionally, Wang *et al.* incorporated H_2_O_2_ enzymes onto semiconductor polymer nanoparticles to enhance tumor oxygenation through situ catalysis, effectively penetrating deep into tumor stroma.^104^

Stimulators of interferon genes (STING) agonists have emerged as promising candidates for cancer immunotherapy due to their broad-spectrum activity and high efficiency.^105^ Zhen *et al.* designed semiconductor polymer nanoagonists (SPNM), in which a photosensitive semiconductor polymer was linked to the STING agonist MSA-2 *via*^1^O_2_-cleavable diphenyl vinylene bonds. This self-assembling nanostructure, SPNM, enabled the *in situ* release of MSA-2 at the tumor site, significantly activating the STING pathway and enhancing the phosphorylation of TBK1 and IRF3, thereby promoting immune cell activation. When combined with the US, SPNM demonstrated significant growth inhibition effects on both primary and distant tumors in SCC-7 tumor-bearing mice, inducing long-term immune memory, as shown in Fig. 6D.^106^ Similarly, Li *et al.* from Donghua University developed sound-activated semiconductor polymer nano-adjuvants (SPN_Ti_) to enhance ICD and suppress myeloid-derived suppressor cells (MDSCs). SPNTi featured a shell formed by ^1^O_2_-cleavable amphiphilic polymers, encapsulating a photosensitive semiconductor polymer, tirapazamine (TPZ) conjugate and MDSC-targeting drug (ibrutinib). Under US irradiation, SPN_Ti_ efficiently generated ^1^O_2_, which facilitated the released of TPZ conjugate and ibrutinib. The continuous depletion of oxygen activated of TPZ for chemotherapy, while ibrutinib inhibited hypoxia-induced MDSC recruitment, promoting ICD. *In vitro* studies confirmed that SPN_Ti_ efficiently produced ^1^O_2_ under US irradiation, leading to tumor inhibition in a bilateral tumor mouse model and effectively preventing tumor metastasis, as shown in Fig. 6E.^107^ These studies provided a theoretical basis for the application of using semiconductor polymers as sonosensitizers and offer valuable insights for developing new sonosensitizers to precisely activate cancer immunotherapy.

### 3.3 Inorganic–organic hybrid sonosensitizers

Although both organic and inorganic nanomaterial sonosensitizers offer distinct advantages, increasing attention has been directed toward organic/inorganic hybrid sonosensitizers to overcome the limitations of each type. Organic/inorganic hybrid sonosensitizers can be primarily categorized into two types: the first involves the direct hybrid coupling of organic and inorganic materials, which can be achieved through physical encapsulation or chemical coupling to form nanomaterial-based sonosensitizers. The second type consists of composite sonosensitizers based on MOFs. These hybrid systems combine the unique properties of both organic and inorganic components, offering enhanced performance for SDT (Table 3).

**Table 3:** Categorization of inorganic–organic hybrid sonosensitizers in recent years

Category	Material	Disease model	US parameters	US time	Ref.
Simple hybrid sonosensitizers	MNS–α-amylase/PEG–Ce6 nanosheet	*Staphylococcus aureus*	1 MHz, 1 W cm^−2^	10 min	211
	RB/SNO/Mn^2+^/SiO_2_ NPs	Breast cancer	0.1 MHz, 1 W cm^−2^, 50% duty cycle	10 min	212
	F-MSN-DOX@RBC	Prostatic neoplasms	HIFU: 2 MHz, 50% duty cycle	5 min	213
	PLGA–HMME–DTX@MnO_2_	Breast cancer	1 MHz, 1 W cm^−2^	3 min	214
	CS-ID@NMs	Colon cancer	3 MHz, 1.5 W cm^−2^, 50% duty cycle	10 min	108
	MnSiO_3_–Pt@BSA–Ce6	Cervical cancer	1 MHz, 1.5 W cm^−2^, 50% duty cycle	5 min	215
	HSA–Ce6–IrO_2_ nanosphere	Breast cancer	1 MHz, 1 W cm^−2^, 50% duty cycle	1 min	216
	C–TiO_2_/TPZ@CM	Colon cancer	40 kHz, 3 W cm^−2^, 50% duty cycle	10 min	29
	PTK@PEG/DOX	Breast cancer	1 MHz, 2 W cm^−2^	15 min	217
	TiO_2_@Pt/GOx (TPG)	Breast cancer	1.0 MHz, 1.0 W cm^−2^, 50% duty cycle	5 min	218
	PCN-224@Pt@GOx@EM	Pancreatic cancer	3 MHz, 0.1 W cm^−2^	5 min	219
MOFs-based sonosensitizers	Ce6/chloroquine/Pt bionic polydopamine nanoparticles	Colorectal cancer	1 W cm^−2^	3 min	220
	RB@COFs-MnO_*x*_-PEG	Osteosarcoma	1 MHz, 1 W cm^−2^, 50% duty cycle	5 min	221
	ReCORMs	Breast cancer	1 MHz, 0.3 W cm^−2^	20 min	222
	MnVO_3_	Breast cancer	40 kHz, 3 W cm^−2^, 50% duty cycle	5 min	223
	CPCs@PEG	Breast cancer	1 MHz, 1 W cm^−2^, 60% duty cycle	5 min	224
	Co–CeO_2_@PEG	Breast cancer	1 MHz, 1 W cm^−2^, 50% duty cycle	5 min	225
	Cu-doped polypyrrole	Lung cancer	1 MHz, 1.5 W cm^−2^, 50% duty cycle	5 min	111
	ZIF-90@MnO_2_/DOX	Cervical cancer	1 MHz, 1 W cm^−2^, 50% duty cycle	1 min	226
	Hb@ZIF-8	Breast cancer	1 MHz, 1.5 W cm^−2^, 50% duty cycle	5 min	227
	MnO_2_–Poly(I:C)@COF	Breast cancer	1 MHz, 0.5 W cm^−2^, 50% duty cycle	5 min	228
	AMR-MOFs@AuPt	Liver cancer	40 kHz, 1 W cm^−2^, 50% duty cycle	5 min	229
	Zr-TCPP(TPP)/R837@MOFs	Breast cancer	1 MHz, 1.5 W cm^−2^, 50% duty cycle	1 min	230
MOFs-based sonosensitizers	THPP-Oxa(IV)-PEG	Colon cancer	40 kHz, 2 W cm^−2^	30 min	231
	MOFs/CPT-Azo	Breast cancer	1 W cm^−2^	3 min	232

#### 3.3.1 Simple hybrid sonosensitizers

Research has shown that metal-doped organic monogenistic materials possess significant antitumor properties. Cao *et al.* developed a multifunctional drug delivery system by loading mitochondria-targeted indocyanine green derivatives (IDs) into mesoporous MnO_2_, which were surface-modified with regenerated silk fibroin (RSF) and embedded in chitosan/alginate hydrogels. This system was responsive to pH, ROS, and GSH, showing promise in targeted drug delivery.^108^ Inspired by these findings, several research groups have explored the role of metal-doped organic materials in treating cancers associated with specific pathogens. Enterotoxigenic *Bacteroides fragilis* (ETBF), a key pathogen in the initiation and metastasis of colorectal cancer (CRC) by Qu *et al.* in their study. They constructed an US-responsive albumin-based nanoplatform (Au@BSA–CuPpIX) by co-loading bovine serum albumin (BSA) with copper(ii) protoporphyrin IX (CuPpIX) and Au nanoparticles. The Au@BSA–CuPpIX platform exhibited strong ROS generation under US, demonstrating high SDT efficiency in xenograft and orthotopic colorectal cancer models. Notably, the AuNPs reduced the phototoxicity of metal porphyrins, alleviating severe skin inflammation and damage.^109^ These metal-doped organic photosensitizer-mediated SDT therapies represent promising strategies for treating deep-seated inflammation and solid tumors.^110^ Further innovations in this field have led to the development of Cu-doped poly-pyrrole composites, formed by simple oxidative polymerization and combined with porphyrin to create a composite photosensitizer (CuPPy-TAPP). CuPPy-TAPP exhibited diverse catalytic activities, including the conversion of H_2_O_2_ to O_2_ and ˙OH, as well as the consumption of reducing GSH through the Cu(ii)–Cu(i) transition pathway. This amplification of oxidative stress enhanced the oxidative damage to tumor cells. This nanomedicine demonstrated high tumor specificity, biodegradability, and the ability to promote *in vitro* cell apoptosis and *in vivo* tumor suppression. This innovative approach not only holds promise as a biocompatible anticancer nanocatalyst but also expands the potential for oxidative stress-based cancer therapies.^111^

In addition to single-metal doping, multimetal alloy nanosystems exhibit enhanced stability and catalytic activity due to electronic coordination effect. Cao *et al.* developed a high biocompatible, ultra-small Cu_2_O-coordinated carbon nitride based on cerium dioxide (Cu_2_O–CN_*x*_@CeO_2_) using a self-catalytic growth strategy. This composite demonstrated both oxidase and catalase activities. *In vivo* and *in vitro* studies showed that laser and US could activate Cu_2_O–CN_*x*_@CeO_2_ to catalyze the production of ROS, significantly inhibiting the growth of malignant melanoma. This research offers a novel approach for designing biocatalysts and achieving multi-mode tumor treatments.^112^

#### 3.3.2 MOFs-based sonosensitizers

**Fig. 7:**
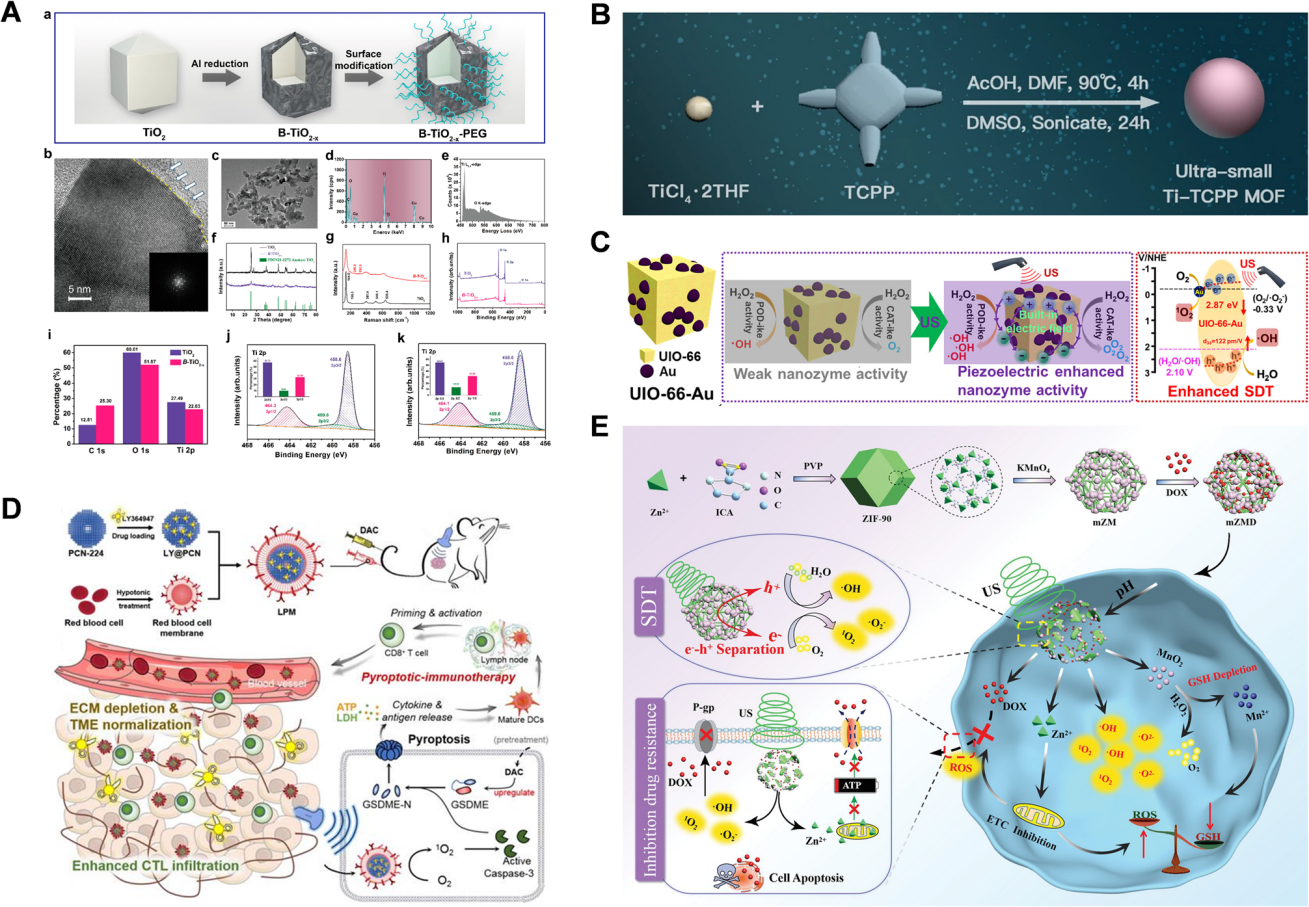
(A) Structural composition and characterization of B-TiO_2−*x*_. (B) Synthesis approach for ultra-small Ti-TCPP MOFs. (C) Fabrication of UIO-66-Au NPs for enhanced piezoelectric SDT and nanozyme catalytic treatment. (D) Diagram illustrating anti-tumor immunotherapy triggered by the sonodynamic-immunomodulatory nanostimulator LPM. (E) Synthesis process and mechanism of mZMD, designed to improve SDT and overcome cancer drug resistance. (A) Reproduced with permission.^117^ Copyright 2018, American Chemical Society. (B) Reproduced with permission.^118^ Copyright 2021, BioMed Central. (C) Reproduced with permission.^124^ Copyright 2023, American Chemical Society. (D) Reproduced with permission.^120^ Copyright 2023, Ivyspring International Publisher. (E) Reproduced with permission.^123^ Copyright 2022, John Wiley & Sons.

MOFs are crystalline, porous materials formed through the self-assembly of transition metal ions and organic ligands, resulting in highly ordered network structures. Due to their simple preparation methods, diverse compositions, large surface area, tunable structures and ease of modification, MOFs exhibit significant potential for development and have been widely applied in the biomedical field.^113^ MOFs contain abundant characteristic nanopores, which can be utilized as precursors or templates for the efficient and controlled preparation of nanostructured derivatives. These frameworks typically feature porous structures with numerous active sites, making them highly versatile. Moreover, by forming MOFs-based nanocomposites, it is possible to reduce the size of the various components to the nanoscale, enhancing their properties through synthetic modifications, functionalization, and other techniques. These modifications can impart unique magnetic, catalytic, and optical properties to MOFs, thereby improving their performance in applications such as SDT.^114^ Consequently, MOFs-based sonosensitizers offer significant advantages, making them highly promising for future biomedical applications.^115,116^ Furthermore, Ti-TCPP-based MOFs, such as defect-rich Ti-based MOFs (D-MOFs(Ti)), have been developed. These MOFs exhibited significant Fenton-like activity and efficient ROS generation under US activation, as confirmed by electron spin resonance (ESR) analysis using 5,5-dimethyl-1-pyrroline *N*-oxide as a spin trap, along with methylene blue degradation experiments. These multifunctional titanium-based photosensitizers show excellent biodegradability and high biocompatibility, making them effective for synergistic SDT and CDT, as shown in Fig. 7A.^117^ Similarly, ultra-small Ti-TCPP-based MOFs exhibit high nuclear targeting and operate independently of the tumor hypoxic microenvironment, as shown in Fig. 7B.^118^ An example is PCN-224, which is formed by coordinating H_2_TCPP with zirconium (Zr) and is loaded with LY364947, a selective inhibitor of TGFβRI.^119^ This nano-formulation effectively prevents collagen deposition and loosens the extracellular matrix structure. *In vivo* studies have demonstrated that this formulation not only enhances T-lymphocyte infiltration but also achieves near-eradication of tumors in a mouse model, promoting immune memory and improving the overall therapeutic outcomes, as shown in Fig. 7D.^120^

Through encapsulation or derivatization, both organic and inorganic sonosensitizers can be incorporated into MOFs-based composite sonosensitizers to enhance their therapeutic efficac.^121^ In the case of organic sonosensitizers, An *et al.* encapsulated nitric oxide glutathione and Ce6 within ZIF-8 and coated the particles with homologous tumor cell membranes, creating a pH/US-responsive biomimetic nanoplatform.^122^ This approach demonstrated promising potential for targeted treatment in the TME. For inorganic sonosensitizers, Guan *et al.* developed a biodegradable composite with doxorubicin (DOX), ZIF-90@MnO_2_/DOX nanoparticles. Under US irradiation, this composite facilitated electron–hole separation and induced cellular oxidative stress. MnO_2_ catalyzed the conversion of endogenous H_2_O_2_ into OH and O_2_, thereby improving the hypoxic TME and enhancing SDT. Additionally, MnO_2_ served as a magnetic resonance imaging contrast agent for real-time therapeutic guidance, combining imaging and treatment into a single platform, as shown in Fig. 7E.^123^ Similarly, UIO-66-Au NPs functioned as piezoelectric sonosensitizers for SDT and US-enhanced catalysis. With a non-centrosymmetric crystal structure, UIO-66-Au NPs generated an intrinsic electric field under US irradiation, facilitating electron–hole separation and ROS production. The incorporation of gold further inhibited electron–hole recombination, thereby enhancing the piezoelectric properties of the materials. In both *in vitro* and *in vivo* experiments, UIO-66-Au NPs-mediated SDT achieved a tumor suppression rate of 93.2%, significantly higher than the 54.1% observed with UIO-66 NPs alone. This study highlighted the synergistic benefits of enhanced SDT and enzyme catalysis, emphasizing the clinical potential of piezoelectric materials and enzymes in oncological therapies, as shown in Fig. 7C.^124^

### 3.4 Other sonosensitizers

**Fig. 8:**
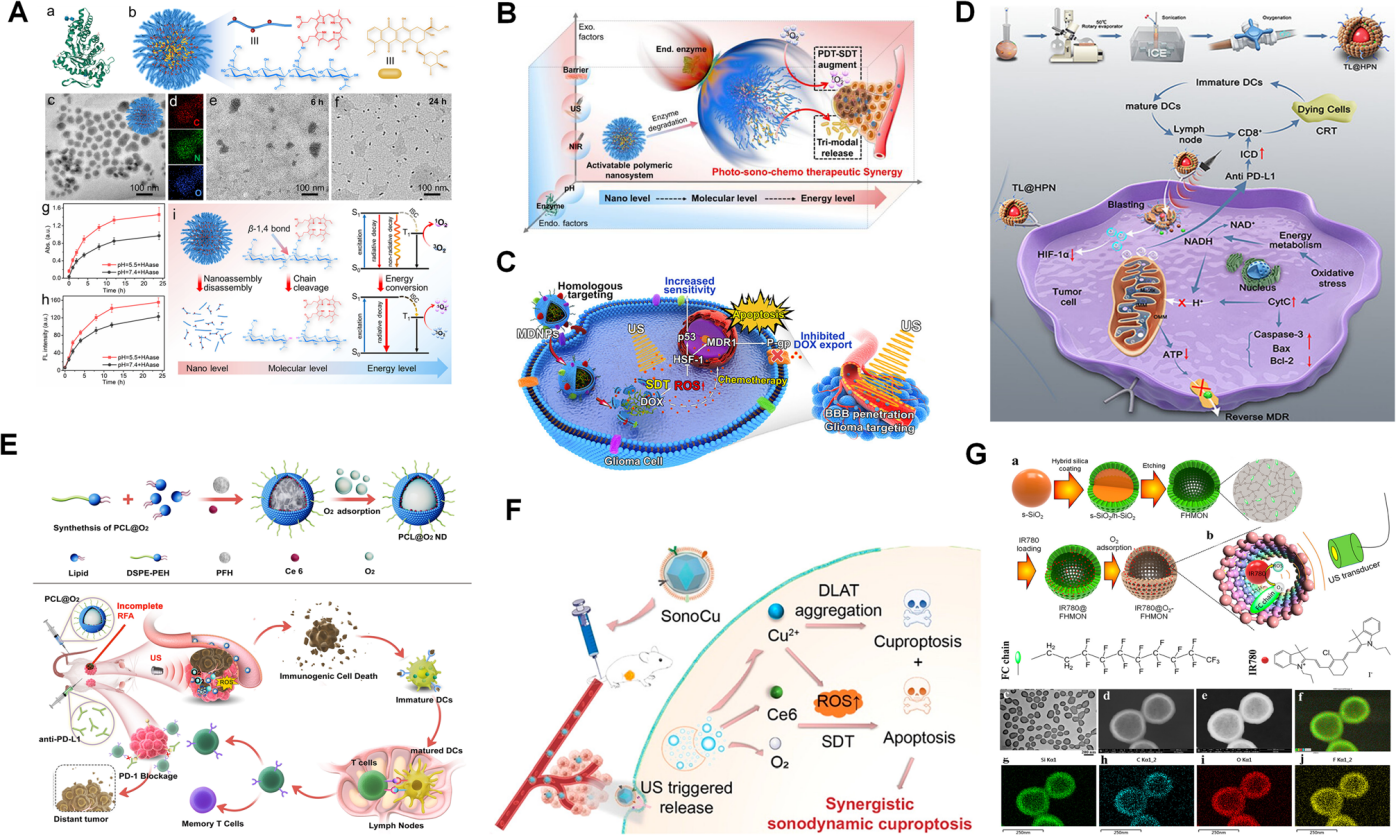
(A) Preparation and degradation of the nanosystem: (a) structure of HAase from bovine testes. (b) DOX@HPNAs polymeric nanosystem comprising HA-PpIX and DOX molecules. (c) TEM image and (d) elemental mapping of HPNAs. (e and f) TEM images of d-HPNAs after enzymatic degradation at 6 and 24 h. Time-dependent changes in (g) absorbance at 400 nm and (h) emission intensity at 630 nm of the supernatant from HPNAs + HAase solutions (pH = 5.5 or 7.4, 37 °C, *n* = 3). (i) Dynamic effects of HAase on HPNAs at nano, molecular, and energy levels. (B) Illustration of the dynamic influences of endogenous (pH, HAase) and exogenous (NIR, US and tissue barriers) factors on activatable polymeric nanosystems (DOX@HPNAs) for achieving photo–sono–chemo synergy in targeting malignant tumors. (C) Conceptual diagram of the design and function of biomimetic sonotheranostic MDNPs. (D) Schematic of US-controlled TL@HPN-mediated mitochondrial energy metabolism disruption, combined with PD-1/PD-L1 modulation to overcome tumor MDR. (E) Diagram illustrating the synthesis of O_2_-loaded PFH–Ce6 liposome@O_2_ nanodroplets (PCL@O_2_) and their *in vivo* anti-tumor performance in enhanced SDT-immune combination therapy. (F) Illustration of SonoCu-mediated synergistic anticancer effects *via* cuproptosis and SDT. (G) Synthesis process and working principle of IR780@O_2_-FHMON and characterization of FHMON carriers: (a) schematic of IR780@O_2_-FHMON. (b) Principle of intensified SDT using IR780@O_2_-FHMON. (c–e) TEM, dark-field, and bright-field images of FHMON carriers. (f–j) Merged and individual atom mapping images (Si (f), C (g), O (h), F (i)) of FHMON carriers. (A and B) Reproduced with permission.^126,129^ Copyright 2022, American Chemical Society. (C) Reproduced with permission.^125,126^ Copyright 2023, American Chemical Society. (D) Reproduced with permission.^133,134^ Copyright 2023, Frontiers MEDIA SA. (E) Reproduced with permission.^112,134^ Copyright 2023, American Chemical Society. (F) Reproduced with permission.^112,135^ Copyright 2017, American Chemical Society. (G) Reproduced with permission.^135,136^ Copyright 2021, Wiley-VCH Verlag GmbH.

Some chemotherapy drugs have shown potential sonosensitizing activity. In one study, researchers encapsulated the chemotherapy drug mitoxantrone (MTX) in hollow Au nanoshells and injected the formulation into mice with breast cancer. The mice were then continuously treated with US at 0.8 MHz and 1.5 W cm^−2^ for five minutes. The result demonstrated that US effectively activated MTX, marking the first exploration of its sonosensitizing activity. Previous *in vitro* studies have also shown that DOX can function as a sonosensitizer, enhancing the antitumor effects of the drug while reducing its side effects. In an *in vivo* studies, researchers utilised polyglutamic acid nanoparticles (MDNPs) encapsulating DOX, which were further coated with human glioblastoma multiforme (GBM) U87 cell membranes. These MDNPs exhibited favorable properties, including homologous targeting, biodegradability and pH sensitivity, thereby improving the therapeutic efficacy of DOX in SDT. Once endocytosed by GBM cells, the membrane-disguised MDNPs rapidly released DOX in the acidic endo/lysosomal environment (pH 5–6), thereby exerting antitumor effects, as shown in Fig. 8C.^125^ Another research group developed hyaluronidase-hyaluronic acid and porphyrin nanosystems loaded with DOX. In this system, the acidic TEM facilitated the enzymatic degradation of hyaluronidase (HAase) on HPNAs, leading to nanoparticle disintegration, drug release, and accumulation of ROS, as shown in Fig. 8A.^126^ Additionally, nanobubbles, lipid-based structures with a gas core, are well-established as US contrast agents in clinical settings. Due to their cavitation effect, nanobubbles have also been explored as sonosensitizers, enhancing the therapeutic efficacy of SDT.^127,128^ For example, combining microbubbles (MBs) with various sonosensitizers has been shown to enhance the performance of SDT. One approach involves integrating carbon dots into the microbubble shell, forming C-dots MBs. This combination allows the carbon dots to effectively absorb energy from inertial cavitation, inducing lipid peroxidation, elevating intracellular ROS, and promoting cell apoptosis, as shown in Fig. 8B.^129^ To extend the application of US contrast agents for deep tissue detection, researchers have developed a range of nanoscale bubbles, such as nano-fluorocarbon emulsions, including perfluorocarbons (PFCs) and perfluorobutane (PFH).^130,131^ These nanoemulsions, which belonged to the PFC family, exhibit liquid–gas phase transition properties, a phenomenon known as acoustic droplet vaporization (ADV). Under US irradiation, PFCs transited from liquid to gas, cavitate and open vascular walls and cell membranes, thereby enhancing tumor cell internalization.^132^ Based on these phase transition properties, research into acoustic nanodroplets has gained momentum. For example, liposomes with a PFP core, loaded with hematoporphyrin monomethyl ether (HMME) and paclitaxel (PTX) and surface-attached with a targeting peptide form a nanosystem called TL@HPN. This system can controllably release drugs and oxygen under US irradiation, alleviating tumor microenvironment hypoxia while sustaining high levels of oxidative stress, which in turn promotes elevated ROS production, as shown in Fig. 8D and Table 4.^133^

**Table 4:** Categorization of other sonosensitizers in recent years

Category	Material	Disease model	US parameters	US time	Ref.
Other sonosensitizers	LIP-PFH	Breast cancer	1 W cm^−2^	2 min	233
	PFH–Ce6 liposome@O_2_ nanodroplets	Colon cancer	1 MHz, 1.6 W cm^−2^, 50% duty cycle	8 min	134
	DET–PpIX–PEG@PFH nanovesicle	Breast cancer	1 MHz, 2.6 W cm^−2^, 25% duty cycle	5 min	234
	IR-780/Gd-DTPA/PFH/@PLGA NPs	Cervical cancer	2.5 W cm^−2^	2–6 min	235
	PFH-C nanosphere		HIFU, 200 W	9 s	236
	C-dots MBs	Prostate cancer	1 W cm^−2^, 50% duty cycle	3 min	129
Other sonosensitizers	5-FU/IRIN/OX/FOL MB	Colorectal tumours	1 MHz, 3.5 W cm^−2^, 30% duty cycle	3.5 min	237
	TL@HPN	Breast cancer	1.6 W cm^−2^, 50% duty cycle	3 min	133
	g-C_3_N_4_/Ce6	Breast cancer	1 W cm^−2^	3 min	238

Similarly, multifunctional nanomedicine, such as PCL@O_2_, have been developed to enhance the thermal ablation effect of radiofrequency ablation by encapsulating Ce6 and PFH droplets as sonosensitizers. PFH not only increased O_2_ content but also possessed self-oxygen enriching capabilities, which prolongs ROS retention within tumors and enhances the efficacy of SDT, as shown in Fig. 8E.^134^ To overcome the limitations of PFC compounds, such as poor stability and restricted application, they can be functionally modified. Conjugating PFCs with targeted functional groups or other materials can optimize their properties and functionalities, thereby enhancing their sensitizing and therapeutic effects. For example, to improve biocompatibility and stability, researchers developed a nanocomposite called SonoCu, composed of macrophage membrane-coated ZIF-8, PFC and Ce6. SonoCu exhibited US-responsive cytotoxicity selectively towards cancer cells and synergized with copper-induced cell death mechanisms to enhance SDT. This study marked the first integration of SDT with copper-induced cell death for cancer treatment, driving further research into rational multimodal therapeutic strategies, as shown in Fig. 8F.^112^ To further increase functionality, another study combined functionalized fluorocarbon (FC) chains with hollow mesoporous organosilica nanoparticles, which serving as carriers of oxygen reservoirs and IR780. The self-oxygen-generating nanoplatforms, IR780@O_2_-FHMON, demonstrated high cytotoxicity and tumor inhibition rates *in vitro* and *in vivo*. This system effectively alleviating hypoxia in PANC-1 solid tumors and overcoming hypoxia-specific transport barriers. This unique FC-chain-mediated oxygen delivery method holds significant potential for mitigating therapy resistance caused by tumor hypoxia, as shown in Fig. 8G.^135^

## 4. Current status and challenges of clinical translation of SDT

In recent years, SDT has gradually transitioned from preclinical research to early-stage clinical evaluation, reflecting its growing recognition as a potential non-invasive cancer treatment modality. Several clinical trials have been conducted to assess the safety, feasibility, and preliminary efficacy of SDT, particularly in gliomas and breast cancer.^137,138^ These studies have primarily utilized porphyrin derivatives such as hematoporphyrin,^139^ Photofrin,^140^ or 5-aminolevulinic acid (5-ALA)^141,142^ as sonosensitizers, due to their previously established clinical safety in PDT. For example, researchers conducted a clinical study on patients with recurrent glioblastoma, in which hematoporphyrin-based SDT combined with focused US resulted in partial tumor necrosis and prolonged progression-free survival without significant neurotoxicity.^143,144^ Similarly, a phase I/II clinical trial using 5-ALA-mediated SDT in glioma patients demonstrated promising safety outcomes, with most patients tolerating the treatment well and exhibiting localized tumor control.^145–148^ In another exploratory study, porphyrin-based SDT for pancreatic cancer showed mild and transient local tissue responses without severe systemic toxicity, suggesting that SDT could be applied even to deep-seated tumors under optimized US exposure conditions.^149–151^ From a pharmacokinetic perspective, the systemic distribution and tumor accumulation of clinically used sonosensitizers are critical to achieving therapeutic efficacy.^152,153^ In these clinical studies, intravenous administration has been the most common route, enabling sufficient tumor uptake through the enhanced permeability and retention (EPR) effect.^154^ Moreover, the development of image-guided detection tools, such as photoacoustic and fluorescence imaging, has facilitated real-time monitoring of sonosensitizer biodistribution and determination of the optimal US exposure time window for SDT activation.^155,156^ Despite these encouraging results, several challenges remain to be addressed before SDT can be widely adopted in clinical oncology. First, the lack of standardized US parameters (frequency, intensity, duty cycle, and exposure duration) across studies makes cross-comparison and reproducibility difficult.^13,157^ Second, the precise mechanisms of SDT in human tissue, particularly regarding intramembrane cavitation and ROS formation, require further elucidation under clinically relevant conditions.^158^ Third, while most clinical trials have reported mild local inflammation or transient fatigue as the major adverse events, the long-term biosafety, systemic immune responses, and pharmacokinetics of emerging nanosonosensitizers have not been fully investigated.^159^ Therefore, future clinical translation should focus on: (i) developing clinically approved nanosonosensitizers with well-defined metabolic and excretion pathways; (ii) optimizing non-invasive imaging tools to guide real-time SDT treatment planning; and (iii) establishing standardized treatment protocols for consistent and reproducible outcomes. With continuous advancements in sonosensitizer engineering, US technology, and image-guided therapy, SDT is expected to evolve into a clinically viable and safe therapeutic approach for cancer patients in the near future.

## 5. Outlook

US has become a very promising detection approach due to its deep penetration, non-invasive and targeted nature and US-mediated SDT also become a very promising treatment method.^160–162^ The therapeutic efficacy of SDT is highly dependent on the nature of the sonosensitizers.^6^ Sonosensitizers have a wide range of biomedical applications in multiple fields, including mediating disease treatment, bioimaging and drug delivery. Therefore, it is crucial to develop sonosensitizing materials that have better performance, activity, and safety. The rapid development of nanobiotechnology has facilitated the exploration and innovation of novel sonosensitizers based on nanoplatforms. Although the research on sonosensitizers made some progress in recent years, there were still some deficiencies, and there were still some key issues to be solved in future clinical applications and treatment modalities.^33,85,113^

(1) Product development and application: although a large number of studies have demonstrated that sonosensitizers-mediated SDT can effectively inhibit tumor growth in a hormonal mouse model, showing its effectiveness and achieving effects such as clearing primary tumors, inhibiting tumor metastasis, and even forming a long-term immune response of the body, most of these studies have remained in the laboratory stage, and the clinical studies and applications have been slow. One of the important reasons is the insufficient assessment of biosafety. Although some studies have provided data on *in vivo* metabolism, long-term toxicity and pharmacokinetics, they remain insufficiently comprehensive. To accelerate the translation of SDT from the laboratory to clinical practice, future research should prioritize enhancing the stability and biocompatibility of the materials, minimizing the toxic side effects of sonosensitizers on normal tissues and organs, and thoroughly evaluating their biological effects. Through comprehensive biosafety evaluation and optimization, SDT is expected to become a reliable clinical cancer treatment.

(2) Understanding the mechanism of action: although there have been many explorations of the mechanism of action of sonosensitizers-mediated SDT, it is generally accepted that sonosensitizers are able to absorb the energy of US and generate cytotoxic ROS through a series of reactions, which is the most recognized and widely researched mechanisms at present. However, the specific physical or chemical reaction pathways have not yet been most clearly characterized. Therefore, exploring and elucidating the exact mechanism of action or triggering mechanism of sonosensitizers is important to address the intrinsic problems (*e.g.*, efficiency) of SDT and to advance the translation of nano-sonosensitizers and SDT to the clinic. The study of organic nano-sonosensitizers and their acoustic dynamics involves multiple disciplinary fields such as chemical synthesis, photophysical chemistry, materials science, biological science, biomedical science, biomedical science and biomedical science *etc.* The development of SDT nano-sonosensitizers requires the design and development of more nano-sonosensitizers, relying on the excellent platform of nano-materials science and the establishment of a more efficient and rigorous therapeutic evaluation system in order to achieve better clinical results. Through multidisciplinary collaboration and innovation, it is expected to fundamentally improve the therapeutic efficiency and safety of SDT and promote its application in the clinic.

(3) Relevant diagnostic and therapeutic tools: the therapeutic effect of SDT is closely related to the US conditions (including frequency and intensity). The ideal instrument not only meets the therapeutic demand but also has the functions of imaging and real-time monitoring, so as to timely adjust the drug delivery program, obtain the best therapeutic effect, and realize the integration of diagnosis-detection-treatment. Currently, a very limited number of US devices had been applied in clinical practice, such as ultrasonic knife (also known as high-intensity focused US), low-intensity pulse therapy instrument and so on. In order to promote clinical translation, it is necessary to design and develop appropriate clinical US devices. These devices should be able to provide precise control of US parameters, as well as integrated imaging and monitoring functions to improve the accuracy and effectiveness of treatment, thus promoting the application of SDT in the clinic.

(4) Limitations of existing sonosensitizers: first, the selection and development of sonosensitizers is still in its infancy. Current sonosensitizers, such as indocyanine green and porphyrin analogues, have shown therapeutic effects to a certain extent. Still, their photostability and sonosensitization efficiency are relatively low, making it difficult to achieve efficient tumor ablation *in vivo*. Second, the biodistribution and metabolic pathways of most of the sonosensitizers reported in the study *in vivo* are unclear, which may lead to nonspecific toxicity and side effects during treatment. In addition, the delivery and targeting ability of sonosensitizers still needs to be improved. Most of the existing sonosensitizers enter the body through intravenous or local injection, but their accumulation efficiency at the tumor site is low, leading to unsatisfactory therapeutic effects. How to design sonosensitizer carriers that can target tumor tissues efficiently is an urgent problem that needs to be solved.

In conclusion, SDT based on sensitizing materials has important significance and broad application prospects in tumor therapy. Through these summaries and presentations, we believed that this review would provide new ideas for future research and to promote the application and development of SDT and sonosensitizing materials in the clinic.

## 6. Conclusions

**Fig. 9:**
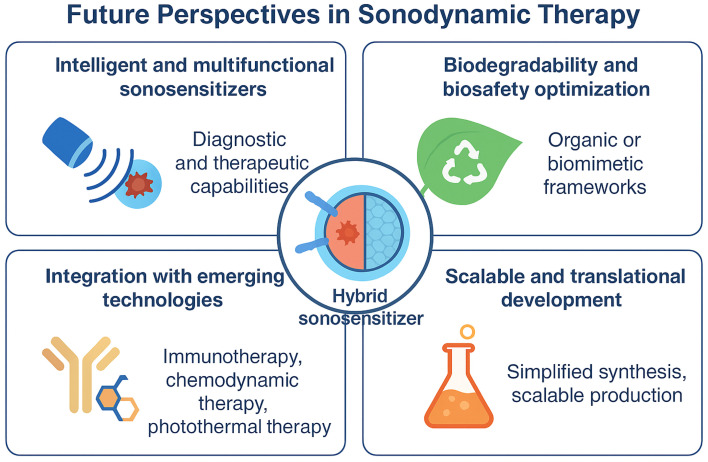
Schematic illustration of future research directions and emerging trends in SDT.

In this review, we introduced the basic principles underlying the ultrasonic cavitation effect and the possible mechanisms of SDT. It also summarized the recent research progress of sonosensitizers, and introduced different types of sonosensitizing materials, mainly including inorganic sonosensitizers, organic sonosensitizers and inorganic–organic hybrid sonosensitizers. However, the clinical translation of SDT still needs to be explored further, including finding more highly efficient and low-toxicity sonosensitizers, exploring multifunctional nanocarriers and investigating the metabolic pathways and biocompatibility of sonosensitizers and so on. Through constant endeavour, SDT is expected to become a safe and effective therapeutic modality of cancer treatment in the future. And more importantly, compared with prior reviews that mainly focused on specific classes of sonosensitizers, this work integrates mechanistic understanding, material innovation, and translational perspectives into a single framework. By discussing inorganic, organic, and hybrid sonosensitizers together with their biomedical implications, this review aims to provide a holistic understanding of SDT and inspire new design strategies for next-generation therapeutic nanoplatforms. In summary, the unique contributions of our review are as follows: (1) comprehensive categorization of all major types of nanobiotechnology-based sonosensitizers, including inorganic, organic, and hybrid systems, rather than focusing on a single material class. (2) Integration of fundamental principles, design rationale, and translational challenges of SDT within one framework, offering both mechanistic depth and practical relevance. (3) Forward-looking perspective, introducing a schematic summary of emerging research directions and clinical translation pathways for SDT-based nanomedicine. Looking ahead, to further advance the clinical translation and therapeutic efficacy of SDT, several key research directions and challenges should be addressed in future studies. (1) Design of intelligent and multifunctional sonosensitizers: future research should focus on developing smart hybrid systems that combine diagnostic and therapeutic capabilities (theranostics), enabling real-time monitoring of SDT efficacy and controlled drug release under ultrasound stimulation. (2) Biodegradability and biosafety optimization: to facilitate clinical translation, more attention should be given to constructing biodegradable, metabolizable, and immunocompatible sonosensitizers based on organic or biomimetic frameworks. (3) Integration with emerging technologies: combining SDT with other modalities-such as immunotherapy, chemodynamic therapy, or photothermal therapy-offers promising avenues for synergistic effects and personalized treatment strategies. (4) Mechanistic insights and computational modeling: advanced imaging, molecular simulations, and theoretical modeling can help elucidate the complex mechanisms of ROS generation and energy transfer, guiding the rational design of next-generation sonosensitizers. (5) Scalable and translational development: emphasis should also be placed on simplifying synthesis routes, improving reproducibility, and adopting scalable fabrication technologies suitable for clinical-grade production. This Fig. 9 is intended to provide readers with an at-a-glance overview of the future development roadmap for SDT.

## Consent for publication

All authors agreed to publish this research in journals.

## Author contributions

Zhaokui Zeng and Tian Tian have equal contributions: conceptualization, methodology, software, data curation, validation, visualization, writing – original draft, editing & revising. Jia Liu: investigation, formal analysis, data curation. Letian Bai: investigation, formal analysis, data curation. Jun Zhang: investigation, formal analysis, data curation. Changhong Nie: formal analysis, data curation, conceptualization. Bo Liu: formal analysis, data curation, conceptualization. Chuanpin Chen and Wei Lu: conceptualization, funding acquisition, supervision, methodology, resources, writing – review & editing.

## Conflicts of interest

The authors declare no conflict interests.

## Data Availability

Data are available from the authors upon request.
